# Supplementary material for: Machine learning reveals distinct neuroanatomical signatures of cardiovascular and metabolic diseases in cognitively unimpaired individuals
Source: Nat Commun. 2025 Mar 19;16:2724. doi: 10.1038/s41467-025-57867-7 (PMC11923046; doi:10.1038/s41467-025-57867-7)
Supplement: Supplementary file 1 — Supplementary Information [file 41467_2025_57867_MOESM1_ESM.pdf]

Supplementary information

Machine learning reveals distinct neuroanatomical signatures of cardiovascular and metabolic diseases in cognitively unimpaired individuals

Sindhuja Tirumalai Govindarajan, PhD [1*], Elizabeth Mamourian, MS [1], Guray Erus, PhD [1], Ahmed Abdulkadir, PhD [2], Randa Melhem, MA [1], Jimit Doshi, MS [1], Raymond Pomponio, BS[1], Duygu Tosun, PhD [3], Murat Bilgel, PhD [4], Yang An, PhD [4], Aristeidis Sotiras, PhD [5], Daniel S. Marcus, PhD [5], Pamela LaMontagne, PhD [5], Tammie L.S. Benzinger, MD, PhD [5], Mark A. Espeland, PhD [6,7], Colin L. Masters, MD [8], Paul Maruff, PhD [8], Lenore J. Launer, PhD [9], Jurgen Fripp, PhD [10], Sterling C. Johnson, PhD [11], John C. Morris, MD [12], Marilyn S. Albert, PhD [13], R. Nick Bryan, MD [14], Susan M. Resnick, PhD [4], Mohamad Habes, PhD [15], Haochang Shou, PhD [1,16], David A. Wolk, MD [17], Ilya M. Nasrallah, MD, PhD [1,14], Christos Davatzikos, PhD [1*,#]

[1] Center for Biomedical Image Computing and Analytics, University of Pennsylvania, Philadelphia, PA, USA

[2] Centre for Artificial Intelligence, ZHAW School of Engineering, Winterthur, Switzerland

[3] Department of Radiology and Biomedical Imaging, University of California, San Francisco, San Francisco, CA, USA

[4] Laboratory of Behavioral Neuroscience, National Institute on Aging, National Institutes of Health, Baltimore, MD, USA

[5] Department of Radiology, Washington University School of Medicine, St. Louis, MO, USA

[6] Sticht Center for Healthy Aging and Alzheimer's Prevention, Wake Forest School of Medicine, Winston-Salem, NC, USA

[7] Department of Biostatistics and Data Science, Wake Forest School of Medicine, Winston-Salem, NC, USA

[8] Florey Institute, The University of Melbourne, Parkville, VIC, Australia

[9] Neuroepidemiology Section, Intramural Research Program, National Institute on Aging, Bethesda, Maryland, USA

[10] CSIRO Health and Biosecurity, Australian e-Health Research Centre CSIRO, Brisbane, Queensland, Australia

[11] Wisconsin Alzheimer's Institute, University of Wisconsin School of Medicine and Public Health, Madison, WI, USA

[12] Knight Alzheimer Disease Research Center, Washington University in St. Louis, St. Louis, MO, USA

[13] Department of Neurology, Johns Hopkins University School of Medicine, Baltimore, MD, USA

[14] Department of Radiology, University of Pennsylvania, Philadelphia, PA, USA

[15] Biggs Alzheimer's Institute, University of Texas San Antonio Health Science Center, San Antonio, TX, USA

[16] Department of Biostatistics, Epidemiology and Informatics, University of Pennsylvania, Philadelphia, PA, USA
[17] Department of Neurology, University of Pennsylvania, Philadelphia, PA, USA

for the iSTAGING study, the Preclinical AD consortium, the ADNI, and the CARDIA studies.
*Corresponding authors

Sindhuja Tirumalai Govindarajan
Email: sindhuja.tirumalaigovindarajan@pennmedicine.upenn.edu

Christos Davatzikos
Email: Christos.davatzikos@pennmedicine.upenn.edu

3700 Hamilton Walk, Richards Building, 7th floor
Center for Biomedical Image Computing Analytics, University of Pennsylvania
19104 Philadelphia, USA
https://www.med.upenn.edu/cbica/

Contents

Abbreviations 4

S1 – iSTAGING Study cohorts 5

S2 – Imaging parameters, image processing and harmonization...6

Supplementary table-1: An overview of magnetic resonance imaging scanners and common parameters of interest from the structural imaging protocols used in individual cohorts. 6

Supplementary table-2: Regions of interest segmented using the multi-atlas, multi-warp segmentation tool (MUSE) 11

2.1 Harmonization of MUSE volumes 15

Supplementary figure-1: Harmonization removes scanner-related variations in ROI volumes 15

S3 – Clinical data consolidation 18

Supplementary table-3: Overview of clinical measures associated with CVMs 19

Supplementary figure-2: Co-occurrence of CVM in the cross-validated training dataset and external testing dataset..20

S4 – Machine learning models 21

4.1 Support vector classification...21

Supplementary figure-3: Study SVC configuration outperforms common machine learning algorithms 22

Supplementary table-4 Additional classification (CVM- and CVM+) performance metrics in the external validation dataset 25

4.2 Age-restricted modeling – Sensitivity analysis 25

S5 – Supplementary Results 26

Supplementary figure-4: Individualized SPARE-CVMs..26

5.1 Spatial patterns of structural changes 27

Supplementary figure-5: Association between SPARE-CVM scores and MRI features........................28

Supplementary figure-6: SPARE-CVM in the external validation cohort 29

Supplementary figure-7: SPARE-CVMs are robust across demographic characteristics 30

Supplementary figure-8: SPARE-CVMs complement established ML-based markers.........................31

Supplementary figure-9: SPARE-CVMs across the age range 32

Supplementary figure-10: Influence of multimorbidity on SPARE-CVMs...33

5.2 Associations with AD pathology 35

Supplementary table-5: Distribution of age, sex, and CVMs in participants with amyloid Aβ data. 35

Supplementary figure-11. Influence of Aβ status, CVM status, and Age on SPARE-CVM scores 36

5.3 Associations between cognitive performance and SPARE-CVM...37

Supplementary table-6: Availability of cognitive test scores 38

Supplementary figure-12: Association between cognitive performance and SPARE-CVMs or CVM status. 39

5.4 Effect of ROI harmonization on SPARE-CVMs...40

Supplementary figure-13: Analysis of Variance (ANOVA) evaluation of site effects 40

Abbreviations

Alzheimer's Disease (AD)
Alzheimer's Disease and related dementias (ADRD)
Alzheimer's Disease Neuroimaging Initiative (ADNI)
Artificial Intelligence (AI)
Australian Imaging, Biomarker, and Lifestyle (AIBL)
Baltimore Longitudinal Study of Aging (BLSA)
Biomarkers of Cognitive Decline Among Normal Individuals (BIOCARD)
Body Mass Index (BMI)
CardioVascular and Metabolic risk factors (CVM)
Coronary Artery Risk Development in Young Adults (CARDIA)
Cognitively Unimpaired (CU)
Digit Symbol Substitution Test (DSST)
Gray Matter (GM)
Hemoglobin A1c (HbA1c)
Hyperlipidemia (HL)
Hypertension (HTN)
Imaging-based coordinate SysTem for AGing and NeurodeGenerative diseases (iSTAGING)
Machine learning (ML)
Magnetic Resonance Imaging (MRI)
Magnetization Prepared-Rapid Gradient Echo (MP-RAGE)
Mild cognitive impairment (MCI)
Mini-mental score examination (MMSE)
Montreal Cognitive Assessment (MOCA)
Obesity (OB)
Smoking (SM)
Structural Magnetic Resonance Imaging (sMRI)
Spatial PAtterns for Recognition of (SPARE-)
Type-2 Diabetes Mellitus (T2D)
UK Biobank (UKBIOBANK)
White Matter Hyperintensities (WMH)
White Matter (WM)
Ventricles (VN)
Wisconsin Registry for Alzheimer's Prevention (WRAP)
+/- notations are the ground truth labels used to indicate the presence/absence of CVM

S1 – iSTAGING Study cohorts

Cohorts used in the iSTAGING study have been previously reported [1] and are briefly outlined below.

The **Alzheimer's Disease Neuroimaging Initiative (ADNI)** [2, 3] is a clinical trial cohort coordinated by the Alzheimer's Therapeutic Research Institute at the University of Southern California. It is recruiting participants from across the United States of America and Canada since the study began in 2004. ADNI recruited over 1,000 subjects with AD, mild cognitive impairment (MCI) and cognitively unimpaired (CU) controls.

The **Australian Imaging, Biomarker, and Lifestyle (AIBL)** [4] study began in Perth and Melbourne, Australia between 2006-2008, as a prospective research study of Alzheimer's Disease, led by the Australian Commonwealth Scientific Industrial and Research Organization3. It focused on subjects above the age of 60 and enrolled 1,112 participants with AD, MCI, and CU.

The **Biomarkers of Cognitive Decline Among Normal Individuals (BIOCARD)** [5] is an ongoing longitudinal study that began in 1995 at the National Institutes of Health. BIOCARD recruited CU adults, 75% with a first-degree relative affected with AD until the study was stopped in 2005. The Johns Hopkins School of Medicine re-initiated the BIOCARD study in 2009, continuing with annual clinical and cognitive follow-up for the original BIOCARD cohort. MR imaging began in 2017 (U19-AG033655).

The **Baltimore Longitudinal Study of Aging (BLSA)** [6] is an ongoing longitudinal study led by the National Institute of Aging (NIA). Established in Baltimore, Maryland, the USA in 1958, BLSA aims to characterize the process of aging, particularly healthy aging. The neuroimaging sub-study of BLSA began in 1994, with annual or semi-annual MRI studies, cognitive testing, and clinical evaluations of a subset of BLSA participants

The **Coronary Artery Risk Development in Young Adults (CARDIA)** [7] study was established in 1984 as a prospective cohort to study the early-life factors that influence the evolution of coronary heart disease. Now in its 35th year, CARDIA has followed its initial cohort from Birmingham, Alabama; Chicago, Illinois; Minneapolis, Minnesota; and Oakland, California at 5-year intervals from recruitment at age 18-30 through adulthood. Starting in year 25, CARDIA began to test cognitive function and acquire brain MRI, as brain aging pathology begins to accumulate during middle-age.

The **Open Access Series of Imaging Studies (OASIS)** [8] aimed at making neuroimaging datasets freely available to the scientific community. By compiling and freely distributing this multi-modal dataset, OASIS hopes to facilitate future discoveries in basic and clinical neuroscience. OASIS-3 is a longitudinal neuroimaging, clinical, cognitive, and biomarker dataset for normal aging and AD.

The **Penn Memory Center (PENN)** [9] participated as an Alzheimer's Disease Research Center (ADRC) in the National Institute on Aging's multi-institutional initiative since 1991. This National Alzheimer's Disease Coordinating Center Uniform Data Set brought together research

being conducted at Penn and 39 other ADRCs from across the USA. Participation in this national initiative, now known at Penn as the Aging Brain Cohort (Penn-ABC) study, requires extensive annual evaluation and examination of the patient's medical, neurological, and psychiatric history. The Penn Memory Center (Penn-PMC) also has a clinical cohort of patients from the Hospital at the University of Pennsylvania, established to complement the Penn-ABC study. The Penn Memory Center administers cognitive testing, including TMT, both to patients participating in the Penn-ABC and Penn-PMC studies.

The **UK Biobank (UKBB)** [10] is a large-scale prospective epidemiologic study, supported by the National Health Service (NHS) since 2006. UK Biobank aims to improve diagnosis, prevention, and treatment of a wide range of diseases by establishing a research cohort of 500,000 participants from across the United Kingdom. The imaging phase of UK Biobank began in 2014 and will include structural and functional brain imaging in 100,000 participants.

The **Women's Health Initiative Memory Study (WHIMS)** [11] acquired brain MRI scans in a subset of 1,403 women aged 71–89 years who participated in an ancillary study to the Women's Health Initiative (WHI).

The **Wisconsin Registry for Alzheimer's Prevention (WRAP)** [12] is a longitudinal observational study of cognitively unimpaired adults. WRAP participants are primarily the healthy children of late-onset Alzheimer's Disease patients at the Memory Assessment Clinic at the University of Wisconsin-Madison.

S2 – Imaging parameters, image processing and harmonization

Supplementary table-1: An overview of magnetic resonance imaging scanners and common parameters of interest from the structural imaging protocols used in individual cohorts.

A subset of studies in the iSTAGING dataset are listed here to showcase the variations in sMRI acquisition protocols. The parameters listed here are representative, and additional information on how scanner variations are encoded in harmonization procedures can be found in Supplementary information S2.1. For more Abbreviations: FLAIR: Fluid-attenuated inversion recovery; MPRAGE: Magnetization-Prepared Rapid Gradient-Echo; PD: Proton density; SPGR: spoiled gradient echo; TSE: Turbo spin echo; TD: Delay time; TE: Echo time; TI: Inversion time; TR: Repetition time

Study	***Scanner***	***T1***	***T2/FLAIR***
**Alzheimer's Disease Neuroimaging Initiative**			
ADNI<br>[2, 3, 13]	3T Siemens	MPRAGE<br>208×240×256 matrix<br>1×1×1 mm^3<br>TE = minimum full echo<br>TR = 2300 ms<br>TI = 900 ms	3D FLAIR<br>256×256×160 matrix<br>1.2×1×1 mm^3<br>TE = 119 ms<br>TR = 4800 ms<br>TI = 1650 ms
	3T Philips	MPRAGE<br>256×240×170 matrix	FLAIR, axial<br>256x237x35 matrix<br>0.86x0.86x5 mm^3

		1×1×1.2 mm^3<br>TE = shortest<br>TR = shortest	TE = 90ms<br>TR = 9000 ms<br>TI = 2500ms<br>———<br>T2-TSE, axial<br>256x255x44 matrix<br>0.94x0.94x4 mm^3<br>TE = 80 ms<br>TR = 3000 ms
	3T GE	SPGR, sagittal<br>256×256 matrix<br>1.2 mm slice thickness<br>Flip angle = 11º<br>TE = minimum full echo<br>TR = 400 ms	FLAIR, axial<br>256×256×42 matrix<br>5 mm slice thickness<br>TE = 147 ms<br>TR = 11000 ms<br>TI = 2250 ms
**Australian Imaging, Biomarker, and Lifestyle**			
AIBL<br>[4, 14]	1.5T<br>Siemens Avanto	MPRAGE, sagittal<br>240×256×160 matrix<br>1×1 mm in-plane<br>1.2 mm slice thickness<br>Flip angle = 9º<br>TE = 2.98 ms<br>TR = 2300 ms<br>TI = 900 ms	T2-TSE, axial<br>228×256 matrix<br>3 mm slice thickness<br>Flip angle = 150º<br>TE = 11 ms<br>TR = 3000 ms
	3T<br>Siemens Trio	MPRAGE<br>160×240×256 matrix<br>1 mm slice thickness<br>Flip angle = 9º<br>TE = 2.98 ms<br>TR = 2300 ms	FLAIR<br>176×240×256 matrix<br>.97 mm slice thickness<br>Flip angle = 120º<br>TE = 421 ms<br>TR = 6000 ms<br>TI = 2100 ms
**Biomarkers of Cognitive Decline Among Normal Individuals**			
BIOCARD [5, 15]	3T<br>Phillips	MPRAGE<br>256×256×170<br>1×1×1.2 mm<br>Flip angle = 8º<br>TE = 3.1 ms<br>TR = 6.8 ms	FLAIR<br>256×256×138<br>1×1×2 mm<br>Flip angle = 90º<br>TE = 100 ms<br>TR = 11000 ms<br>TI = 2800 ms<br>———<br>PD/T2<br>224×224×96<br>.95×.95×1.5 mm<br>Flip angle = 90º

			TE = 28.2 ms<br>TR = 6636.5 ms
**Baltimore Longitudinal Study of Aging**			
	1.5 T<br>GE	SPGR<br>.94×.94×1.5 mm<br>Flip angle = 45º<br>TE = 5 ms<br>TR = 35 ms	PD/T2<br>256 × 192<br>TR = 3000<br>TE = 34/100<br>FOV = 24 cm<br>number of excitations = 0.5
	3T<br>Philips	MPRAGE, sagittal<br>1×1 mm in-plane<br>1.2 mm slice thickness<br>Flip angle = 8º<br>TE = 3.1 ms<br>TR = 6.5 ms or 6.8 ms	T2-FLAIR, axial<br>.8×.8 mm in-plane<br>3 mm slice thickness<br>TE = 68 ms<br>TR = 11000 ms
**Coronary Artery Risk Development in Young Adults**			
CARDIA<br>[7, 16]	3T<br>Siemens Tim Trio (VB17)	MPRAGE, sagittal<br>FOV: 250 mm<br>256×256 matrix<br>1 mm slice thickness<br>Flip angle = 9º<br>TE = 2.89 ms<br>TR = 1900 ms<br>TI = 900 ms	3D FLAIR, sagittal<br>FOV: 250 mm<br>258×221 matrix<br>1 mm slice thickness<br>TE = 160 ms<br>TR = 6000 ms<br>TI = 2200 ms<br>―――<br>3D T2, sagittal<br>FOV: 250 mm<br>258×256 matrix<br>1 mm slice thickness<br>Flip angle = 120º<br>TE = 409 ms<br>TR = 3200 ms
**Open Access Series of Imaging Studies**			
OASIS<br>[8]	1.5T Siemens Vision	MPRAGE, sagittal<br>256x256x128 matrix<br>1.25mm slice thickness<br>Flip angle = 10º<br>TE = 4 ms<br>TR = 9.7 ms<br>TI = 20 ms<br>TD = 200 ms	-
	3T Siemens Biograph	3D MPRAGE<br>256x256x240 matrix<br>1.2mm slice thickness	FLAIR, axial<br>256x256 matrix

		Flip angle = 9º<br>TE = 3 ms<br>TR = 2.3 ms<br>TI = 900 ms<br>TD = 200 ms	5mm slice thickness<br>Flip angle = 150º<br>TE = 91ms<br>TR = 9000 ms<br>TI = 2500 ms
	3T Siemens TrioTim	3D MPRAGE<br>256x256x256 matrix<br>1mm slice thickness<br>Flip angle = 8º<br>TE = 3 ms<br>TR = 2.4 ms<br>TI = 1000 ms<br>TD = 200 ms	FLAIR, axial<br>256x256 matrix<br>5mm slice thickness<br>Flip angle = 120º<br>TE = 76ms<br>TR = 9000 ms<br>TI = 2500 ms
**Penn Memory Center, University of Pennsylvania**			
PENN<br>[9]	3T<br>Siemens Trio	MPRAGE<br>1×1×1 mm<br>———<br>With 8-channel coil:<br>Flip angle = 15º<br>TE = 3.87 ms<br>TR = 1600 ms<br>TI = 950 ms<br>———<br>With 32-channel coil:<br>Flip angle = 9º<br>TE = 2.89 ms<br>TR = 1900 ms<br>TI = 900 ms	T2-TSE<br>.4×.4×2 mm<br>Flip angle = 150º<br>———<br>With 8-channel coil:<br>TE = 68 ms<br>TR = 5310 ms<br>———<br>With 32-channel coil:<br>TE = 76 ms<br>TR = 7200 ms
**UK Biobank**			
UKBB<br>[10, 17]	3T<br>Siemens Skyra (VD13)	MPRAGE, sagittal<br>1×1×1 mm<br>TR = 2000 ms<br>TI = 880 ms<br>TE = 2ms<br>Flip Angle = 8º	FLAIR, sagittal<br>FOV: 256 mm<br>1.05×1×1 mm<br>TR = 5000 ms<br>TE = 395 ms<br>TI = 1800 ms
**Women's Health Initiative Memory Study**			
WHIMS<br>[11]	Multiple (see citation)	3D T1, axial<br>FOV: 220 mm<br>256x256 matrix<br>1.5mm slice thickness<br>Flip angle = 30º<br>TE = 8 ms<br>TR = 21 ms<br>TI = 0 ms	FLAIR, axial<br>FOV: 220 mm<br>256x256 matrix<br>3mm slice thickness<br>TE = 100 ms<br>TR = 8000 ms<br>TI = 2000 ms<br>———

			T2, axial<br>FOV: 220 mm<br>256x256 matrix<br>3mm slice thickness<br>TE = 30, 120 ms<br>TR = 3200 ms<br>TI = 0 ms
**Wisconsin Registry for Alzheimer's Prevention**			
WRAP<br>[12]	3T<br>GE Discovery MR750 (Waukesha, WI, USA) with 8-channel head coil and parallel imaging with Array Spatial Sensitivity Encoding Technique (ASSET)	SPGR, axial<br>FOV: 256 mm<br>156×256 matrix<br>1 mm slice thickness<br>Flip angle = 12º<br>TR = 8.16 ms<br>TI = 450 ms<br>TE = 3.18 ms	3D FLAIR, sagittal<br>FOV: 256 mm<br>256×256 matrix<br>2 mm slice thickness<br>Flip angle = 90º<br>TR = 6000 ms<br>TI = 1868 ms<br>TE = 123 ms

Supplementary table-2: Regions of interest segmented using the multi-atlas, multi-warp segmentation tool (MUSE)

ROIs are categorized under the following tissue groups: GM – gray matter, WM – white matter, and WMH – white matter hyperintensities.

MUSE index #	ROI Name	Tissue Group
23	Right Accumbens Area	GM
30	Left Accumbens Area	GM
31	Right Amygdala	GM
32	Left Amygdala	GM
36	Right Caudate	GM
37	Left Caudate	GM
38	Right Cerebellum Exterior	GM
39	Left Cerebellum Exterior	GM
40	Right Cerebellum White Matter	WM
41	Left Cerebellum White Matter	WM
47	Right Hippocampus	GM
48	Left Hippocampus	GM
55	Right Pallidum	GM
56	Left Pallidum	GM
57	Right Putamen	GM
58	Left Putamen	GM
59	Right Thalamus Proper	GM
60	Left Thalamus Proper	GM
71	Cerebellar Vermal Lobules I-V	GM
72	Cerebellar Vermal Lobules VI-VII	GM
73	Cerebellar Vermal Lobules VIII-X	GM
75	Left Basal Forebrain	GM
76	Right Basal Forebrain	GM
81	frontal lobe WM right	WM
82	frontal lobe WM left	WM
83	occipital lobe WM right	WM
84	occipital lobe WM left	WM
85	parietal lobe WM right	WM
86	parietal lobe WM left	WM
87	temporal lobe WM right	WM
88	temporal lobe WM left	WM
89	fornix right	WM
90	fornix left	WM
91	anterior limb of internal capsule right	WM
92	anterior limb of internal capsule left	WM

93	posterior limb of internal capsule inc. cerebral peduncle right	WM
94	posterior limb of internal capsule inc. cerebral peduncle left	WM
95	corpus callosum	WM
100	Right ACgG anterior cingulate gyrus	GM
101	Left ACgG anterior cingulate gyrus	GM
102	Right AIns anterior insula	GM
103	Left AIns anterior insula	GM
104	Right AOrG anterior orbital gyrus	GM
105	Left AOrG anterior orbital gyrus	GM
106	Right AnG angular gyrus	GM
107	Left AnG angular gyrus	GM
108	Right Calc calcarine cortex	GM
109	Left Calc calcarine cortex	GM
112	Right CO central operculum	GM
113	Left CO central operculum	GM
114	Right Cun cuneus	GM
115	Left Cun cuneus	GM
116	Right Ent entorhinal area	GM
117	Left Ent entorhinal area	GM
118	Right FO frontal operculum	GM
119	Left FO frontal operculum	GM
120	Right FRP frontal pole	GM
121	Left FRP frontal pole	GM
122	Right FuG fusiform gyrus	GM
123	Left FuG fusiform gyrus	GM
124	Right GRe gyrus rectus	GM
125	Left GRe gyrus rectus	GM
128	Right IOG inferior occipital gyrus	GM
129	Left IOG inferior occipital gyrus	GM
132	Right ITG inferior temporal gyrus	GM
133	Left ITG inferior temporal gyrus	GM
134	Right LiG lingual gyrus	GM
135	Left LiG lingual gyrus	GM
136	Right LOrG lateral orbital gyrus	GM
137	Left LOrG lateral orbital gyrus	GM
138	Right MCgG middle cingulate gyrus	GM
139	Left MCgG middle cingulate gyrus	GM
140	Right MFC medial frontal cortex	GM
141	Left MFC medial frontal cortex	GM
142	Right MFG middle frontal gyrus	GM

143	Left MFG middle frontal gyrus	GM
144	Right MOG middle occipital gyrus	GM
145	Left MOG middle occipital gyrus	GM
146	Right MOrG medial orbital gyrus	GM
147	Left MOrG medial orbital gyrus	GM
148	Right MPoG postcentral gyrus medial segment	GM
149	Left MPoG postcentral gyrus medial segment	GM
150	Right MPrG precentral gyrus medial segment	GM
151	Left MPrG precentral gyrus medial segment	GM
152	Right MSFG superior frontal gyrus medial segment	GM
153	Left MSFG superior frontal gyrus medial segment	GM
154	Right MTG middle temporal gyrus	GM
155	Left MTG middle temporal gyrus	GM
156	Right OCP occipital pole	GM
157	Left OCP occipital pole	GM
160	Right OFuG occipital fusiform gyrus	GM
161	Left OFuG occipital fusiform gyrus	GM
162	Right OpIFG opercular part of the inferior frontal gyrus	GM
163	Left OpIFG opercular part of the inferior frontal gyrus	GM
164	Right OrIFG orbital part of the inferior frontal gyrus	GM
165	Left OrIFG orbital part of the inferior frontal gyrus	GM
166	Right PCgG posterior cingulate gyrus	GM
167	Left PCgG posterior cingulate gyrus	GM
168	Right PCu precuneus	GM
169	Left PCu precuneus	GM
170	Right PHG parahippocampal gyrus	GM
171	Left PHG parahippocampal gyrus	GM
172	Right PIns posterior insula	GM
173	Left PIns posterior insula	GM
174	Right PO parietal operculum	GM
175	Left PO parietal operculum	GM
176	Right PoG postcentral gyrus	GM
177	Left PoG postcentral gyrus	GM
178	Right POrG posterior orbital gyrus	GM
179	Left POrG posterior orbital gyrus	GM
180	Right PP planum polare	GM
181	Left PP planum polare	GM
182	Right PrG precentral gyrus	GM
183	Left PrG precentral gyrus	GM
184	Right PT planum temporale	GM
185	Left PT planum temporale	GM

186	Right SCA subcallosal area	GM
187	Left SCA subcallosal area	GM
190	Right SFG superior frontal gyrus	GM
191	Left SFG superior frontal gyrus	GM
192	Right SMC supplementary motor cortex	GM
193	Left SMC supplementary motor cortex	GM
194	Right SMG supramarginal gyrus	GM
195	Left SMG supramarginal gyrus	GM
196	Right SOG superior occipital gyrus	GM
197	Left SOG superior occipital gyrus	GM
198	Right SPL superior parietal lobule	GM
199	Left SPL superior parietal lobule	GM
200	Right STG superior temporal gyrus	GM
201	Left STG superior temporal gyrus	GM
202	Right TMP temporal pole	GM
203	Left TMP temporal pole	GM
204	Right TrIFG triangular part of the inferior frontal gyrus	GM
205	Left TrIFG triangular part of the inferior frontal gyrus	GM
206	Right TTG transverse temporal gyrus	GM
207	Left TTG transverse temporal gyrus	GM
81	frontal lobe WM right - hyperintensities	WMH
82	frontal lobe WM left - hyperintensities	WMH
83	occipital lobe WM right - hyperintensities	WMH
84	occipital lobe WM left - hyperintensities	WMH
85	parietal lobe WM right - hyperintensities	WMH
86	parietal lobe WM left - hyperintensities	WMH
87	temporal lobe WM right - hyperintensities	WMH
88	temporal lobe WM left - hyperintensities	WMH
89	fornix right - hyperintensities	WMH
90	fornix left - hyperintensities	WMH
91	anterior limb of internal capsule right - hyperintensities	WMH
92	anterior limb of internal capsule left - hyperintensities	WMH
93	posterior limb of internal capsule inc. cerebral peduncle right - hyperintensities	WMH
94	posterior limb of internal capsule inc. cerebral peduncle left - hyperintensities	WMH
95	corpus callosum - hyperintensities	WMH

2.1 Harmonization of MUSE volumes

MUSE volumes in the iSTAGING dataset were harmonized using CombatGAM [18], a retrospective harmonization method used to mitigate scanner biases in the features (e.g., ROI volumes) derived from MRI. Scanner-level differences in the manufacturer, magnetic field strength, head coil, gradient and receiving coils, sequence specification, voxel size, etc. are modeled as site-specific differences in mean and variance across the pooled ROI volumes. Corrective site-level shift and scale parameters are then applied to the data to maintain relationships between data points within and across sites/batches.
The term 'site' or 'batch' here refers to the grouping of MRI data acquired using consistent scanner site and parameter settings during study data collection. Studies like ADNI were grouped into 'batch' or 'site' based on their study phase (i.e., ADNI-1, ADNI-2/ADNI-GO, ADNI-3) since the scans were prospectively standardized – i.e., the scanner field strength and sequences were adapted in a data-driven manner to ensure uniformity across the multicenter study [3]. In the case of studies like CARDIA, MRI data collection was performed across three different locations [19], and each is treated as a separate 'batch' or 'site' in ComBat-GAM. Similarly, BLSA-1.5T [20] and BLSA-3T [21] are treated as separate sites in the harmonization model, as the scanning parameters within site/phase were maintained for longitudinal consistency [22].

Supplementary figure-1: Harmonization removes scanner-related variations in ROI volumes

Boxplots below show the volume distributions of specific regions of interest before and after harmonization. ROI measures listed here are residual volumes after correction for age, sex, and intracranial volume. Residuals are grouped by study for the purpose of visualization.

ROI	Pre-harmonization	Post-harmonization

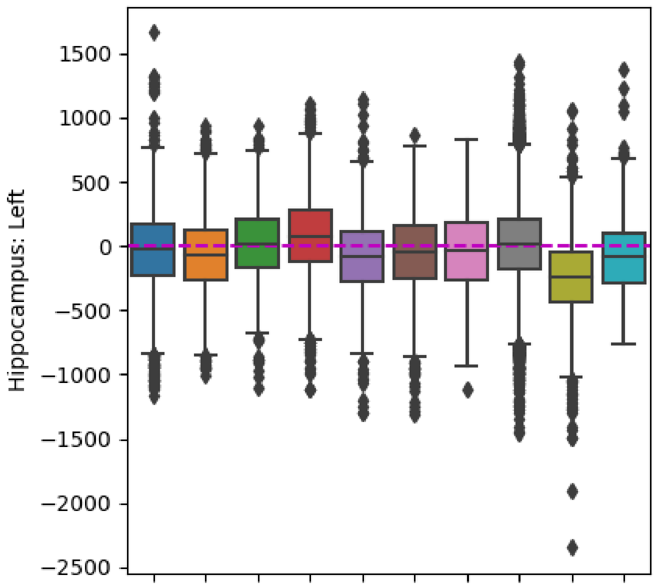

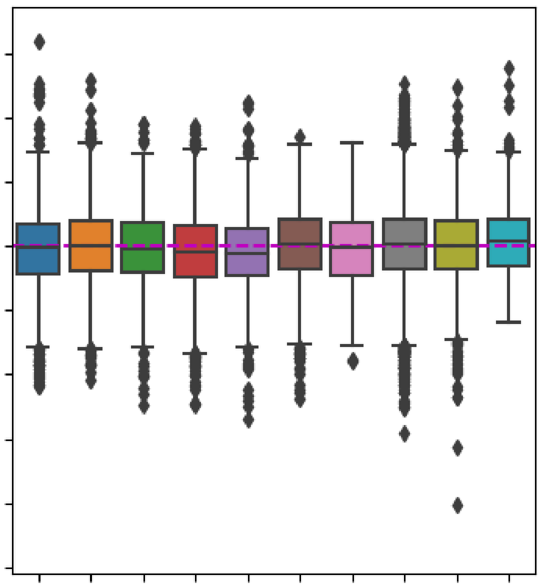

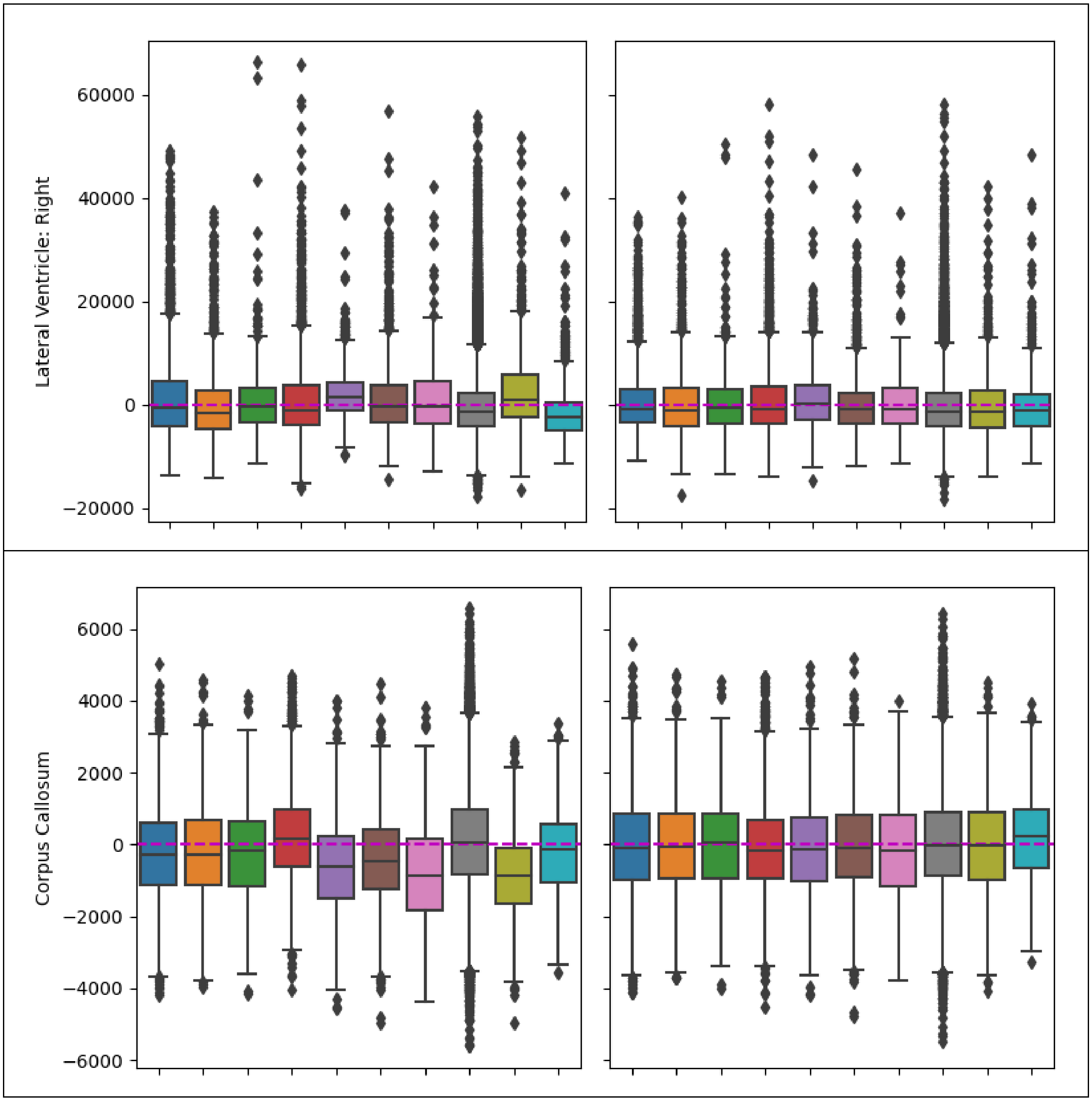

60000
40000
20000
0
−20000
Lateral Ventricle: Right
6000
4000
2000
0
−2000
−4000
−6000
Corpus Callosum

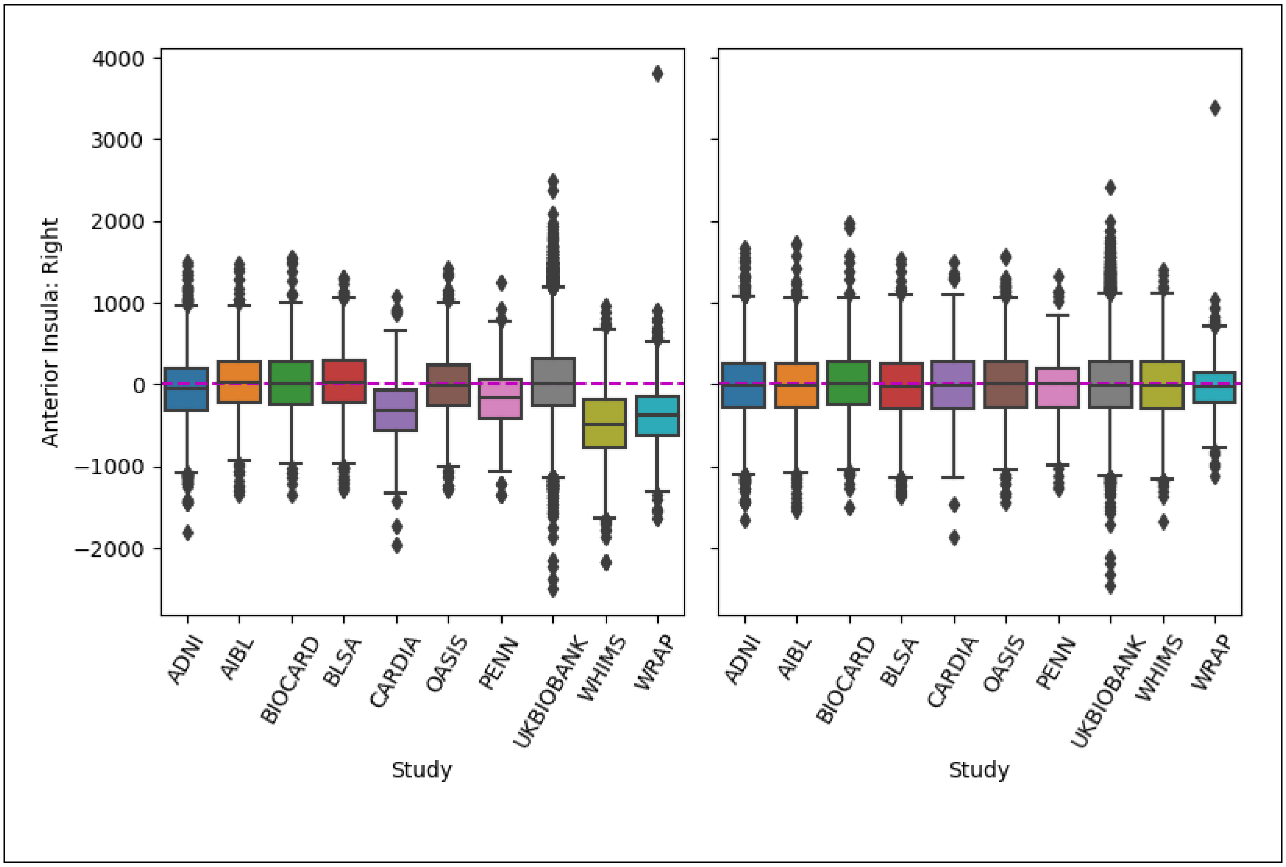
Anterior Insula: Right
4000
3000
2000
1000
0
−1000
−2000
ADNI
AIBL
BIOCARD
BLSA
CARDIA
OASIS
PENN
UKBIOBANK
WHIMS
WRAP
Study
Study

S3 – Clinical data consolidation

Supplementary table-3 below shows an overview of the clinical measures used in dichotomizing CVM status and the availability of these measures in individual cohorts. Briefly, participants study provided (self/physician reports) “Present/Recent” diagnosis or treatment for the corresponding condition were automatically assigned CVM+ status. If participants did not report medical history (missing data), or reported CVM “Not Present”, clinical measures were used in ascertaining CVM status. Participants with continuous measures above the cutoffs indicated under the CVM+ column were assigned CVM+ status. Participants with continuous measures below the cutoffs indicated under the CVM- columns were considered CVM -. If a participant did not have any relevant information for a specific CVM (missing data), they were excluded from the corresponding CVM-related investigations. Participants who reported CVM “Not Present” and yet had continuous measures that were between the CVM- and the CVM+ cutoffs were considered only in validation experiments if the measures could be considered sub-diagnostic (**Main article section**: *Evaluating the associations between SPARE-CVMs and clinical measures of CV health*).

Supplementary table-3: Overview of clinical measures associated with CVMs

Data availability in iSTAGING at the time of the present study are indicated by percentage of participants without null values for the corresponding variable.

Risk factor	Measured variables	Cut-offs		ADNI	AIBL	BIOCARD	BLSA	CARDIA	OASIS	PENN	UKBIOBANK	WHIMS	WRAP
		CVM-	CVM+										
Hypertension	**History of Hypertension**	Not present	Present/Recent	●	◕	◕	●	●	●	◔	◕	◑	●
	Medicated for Hypertension	No	Yes	●	-	●	●	◕	-	-	●	○	-
	Systole	<130 mmHg	>150 mmHg	●	◕	-	●	●	-	○	●	●	-
	Diastole	<80 mmHg	>95 mmHg	●	◕	-	●	●	-	○	●	●	-
	Total N for training	7596	7865										
Hyperlipidemia	**History of Hyperlipidemia**	Not present	Present/Recent	-	◕	◕	○	●	●	◔	◕	◑	●
	Medicated for Hyperlipidemia	No	Yes	●	-	●	-	●	-	-	●	○	-
	LDL	<100 mg/dL	>160 mg/dL	-	-	-	●	●	-	-	●	○	-
	Triglycerides	<130 mg/dL	>200 mg/dL	◑	-	-	●	●	-	-	●	○	-
	Total N for training	7900	6171										
Smoking	**History of Smoking**	Never		◕	◑	◕	●	◑	●	-	●	◑	●
	Years of Smoking	0 years	>= 10 years	◔	◔	◑	-	◔	◑	○	◔	-	-
	Total N for training	10634	4018										
Obesity	**Body mass index**	20-25 kg/m2	>30 kg/m2	●	◕	◕	●	●	●	◔	●	●	●
	Total N for training	7085	4126										
Diabetes	**History of Type 2 Diabetes**	Not present	Present/Recent	◑	◕	◕	○	●	●	◔	●	●	●
	Medicated for Type 2 Diabetes	No	Yes	●	-	●	-	●	-	-	●	○	-
	Glucose	<100 mg/dL	>= 126 mg/dL	◑	◑	-	-	●	-	-	◕	○	-
	HBA1C	<5.7 %	>=6.5 %	-	-	-	-	◕	-	-	●	-	-
	Total N for training	17151	1511										

Key	○	< 20 %	◔	21-40 %	◑	41-70 %	◕	71-90 %	●	> 90 %

Supplementary figure-2: Co-occurrence of CVM in the cross-validated training dataset and external testing dataset.

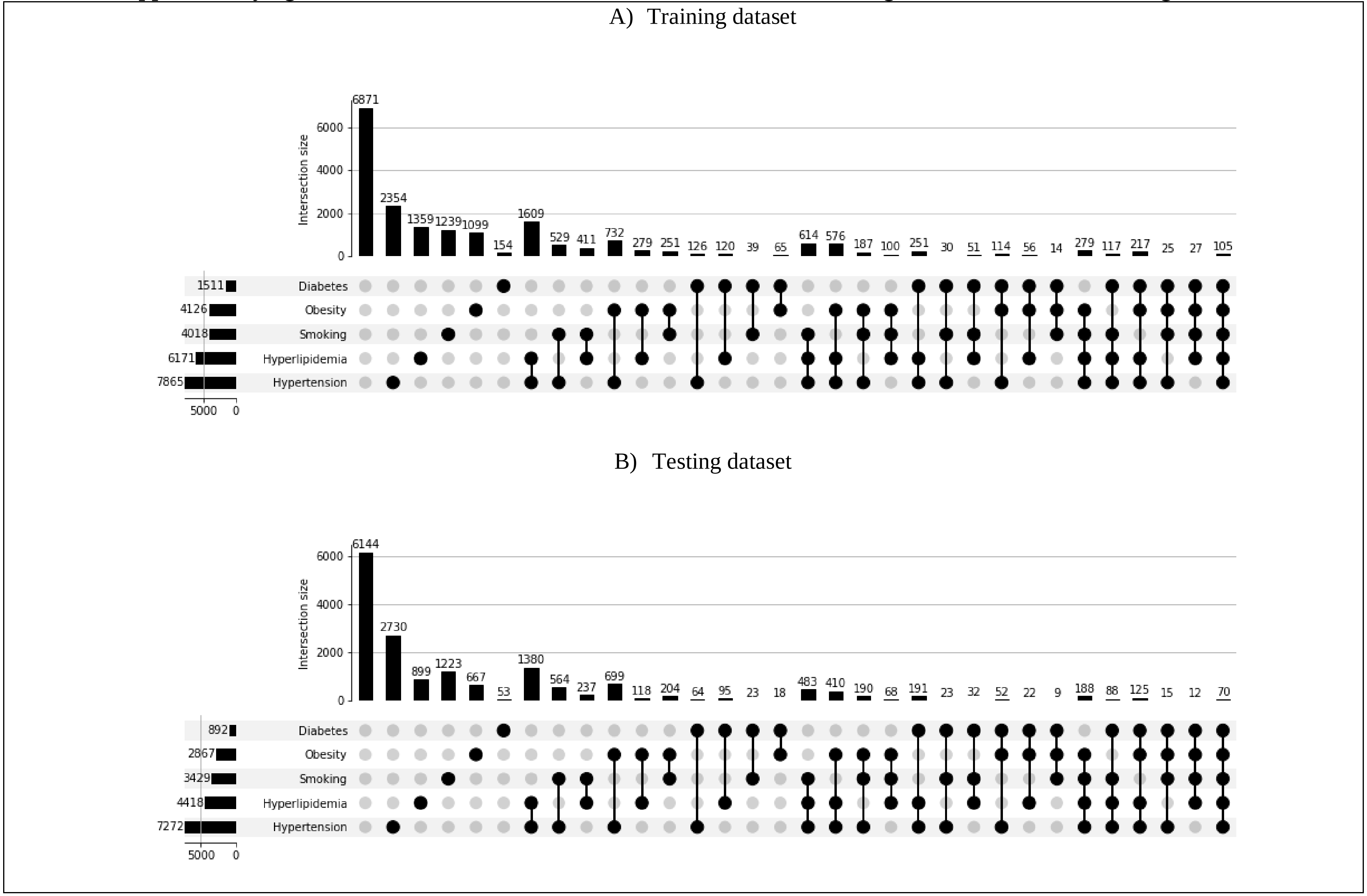

S4 – Machine learning models

4.1 Support vector classification

Support vector classification models are well suited for our study goals for the following reasons: i) SVMs model a hyperplane to maximize the margins between classes and are less susceptible to outliers in the data, ii) are effective with high-dimensional features and large datasets, and iii) SVMs have been robustly utilized in neuroimaging studies using multi-modal imaging data and have shown better performance for detecting Alzheimer's disease [23] [24], Parkinson's disease [25], schizophrenia [26], and other neurological and neuropsychiatric disorders [27].

Linear support vector classifiers from scikit-learn [28] were used in this study to derive models for each CVM separately. A nested cross-validation procedure with k=5 inner and k=10 outer folds was employed to prevent information "leak" between the training labels and test outputs [29]. In other words, the entire dataset was split into k=10 outer-folds, and the training data in each outer-fold was further split into k=5 inner-folds for training and validation. Hyperparameter C was tuned in the inner folds for best average performance, and the average generalization error was estimated on the outer folds using test data which were not part of the training. Class imbalance, i.e., more CVM- than CVM+ samples, was addressed by: i) stratifying the k-folds were stratified to ensure equivalent distribution of classes across each fold, and ii) evaluating model performance using scikit-learn's balanced accuracy scorer which weights raw accuracy by the inverse of class prevalence. The resulting distance of the projection of the test data onto the vector perpendicular to the decision plane yields a continuous scalar representing 'spatial pattern of abnormality for recognition' (SPARE) of each CVM. SPARE-CVMs for external validation data were derived using an ensemble approach, i.e., an average of predictions using all inner models.

To validate the efficacy of our model selection, we conducted an automated machine learning (AutoML) experiments using the same dataset. We employed Auto-Sklearn [30], which explores different hyperparameter configurations for various classification algorithms from scikit-learn [28]. As shown in **Supplementary figure-3**, our SVM implementation achieved superior performance (balanced accuracy and AUC) compared to other models across the outer k-folds.

Supplementary figure-3: Study SVC configuration outperforms common machine learning algorithms

Performance evaluation of our support vector classifier model configuration in comparison with other machine learning models. Metrics shown below are the area under the curve of the receiver operating characteristic (ROC AUC) and balanced accuracy evaluated on the "test" set across each outer fold of the nested cross-validation. Models compared across the outer folds: Support vector classifiers used in the study (K-Nearest Neighbors, Random Forest Classifier, Linear Discriminant Analysis (lda), Quadratic Discriminant Analysis (qda), Multi-layer Perceptron (mlp), Passive Aggressive Classifier (passive_aggressive), Decision Tree Classifier (decision_tree), Extra Trees Classifier (extra_tree), Multinomial Naive Bayes (multinomial-nb), Gaussian Naive Bayes (gaussian_nb), Bernoulli Naive Bayes (bernoulli_nb) AdaBoost Classifier, Gradient Boosting Classifier, and Stochastic Gradient Descent Classifier (sgd).

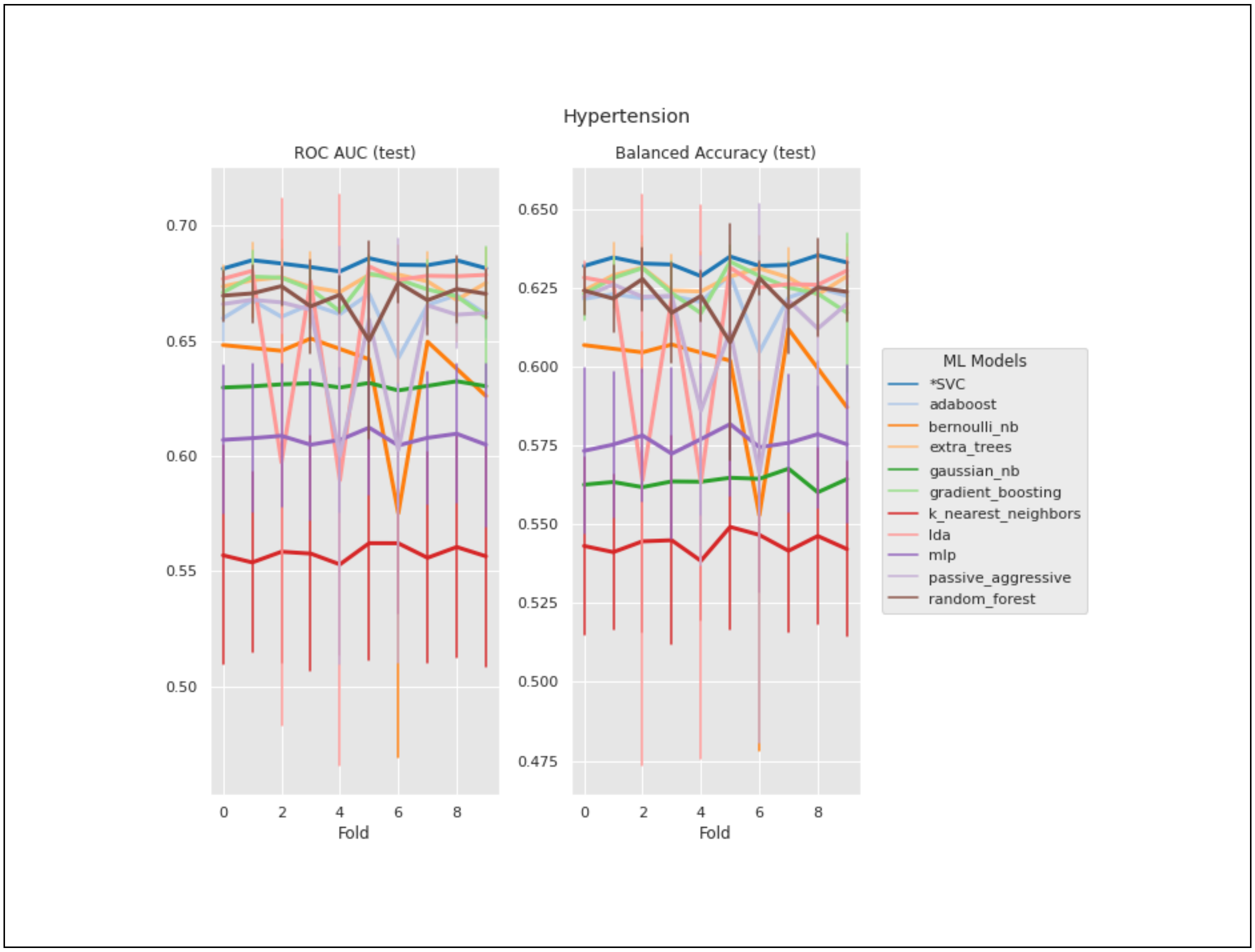

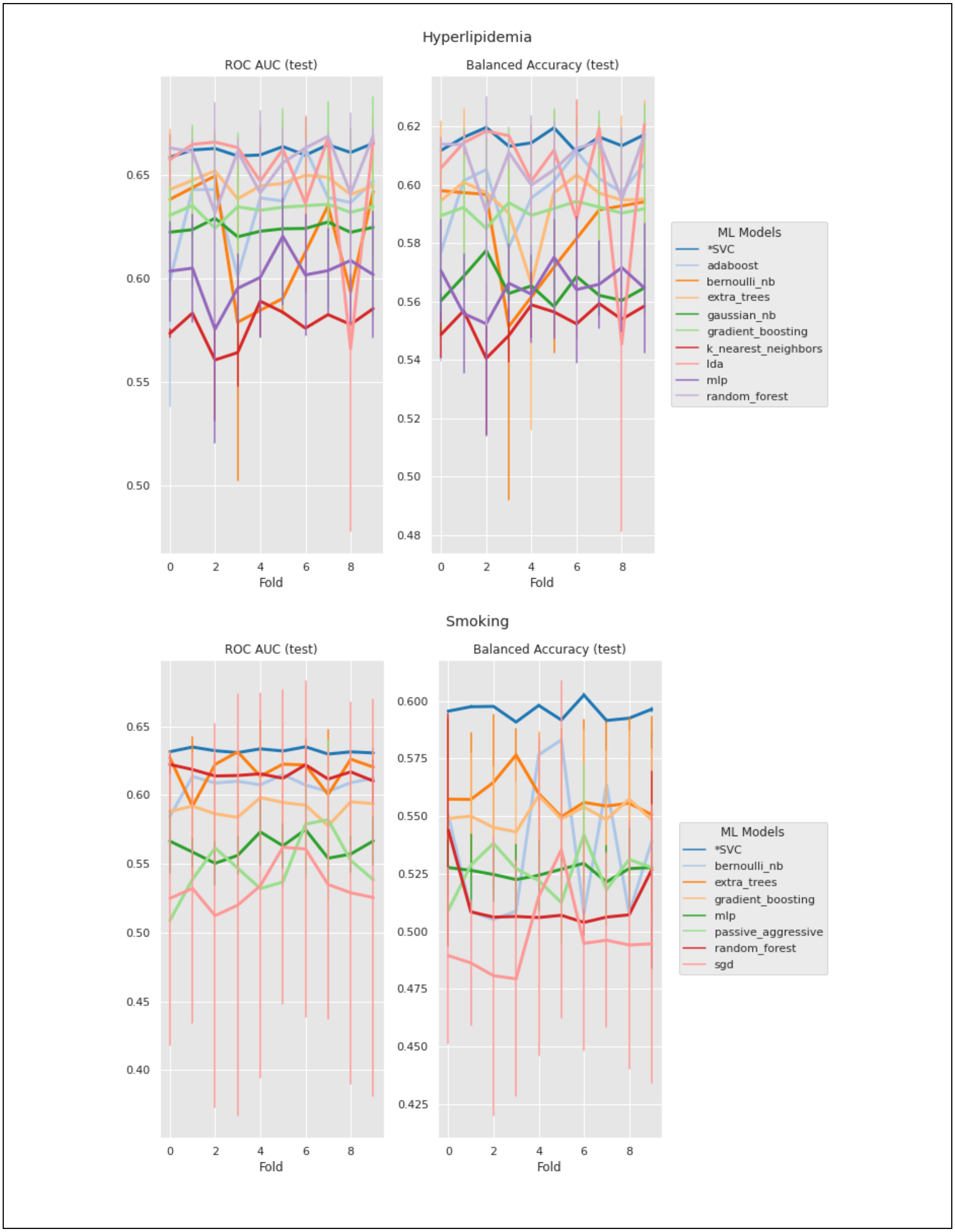
Hyperlipidemia
ROC AUC (test)
Balanced Accuracy (test)
Fold
ML Models
*SVC
adaboost
bernoulli_nb
extra_trees
gaussian_nb
gradient_boosting
k_nearest_neighbors
lda
mlp
random_forest
Smoking
ROC AUC (test)
Balanced Accuracy (test)
Fold
ML Models
*SVC
bernoulli_nb
extra_trees
gradient_boosting
mlp
passive_aggressive
random_forest
sgd

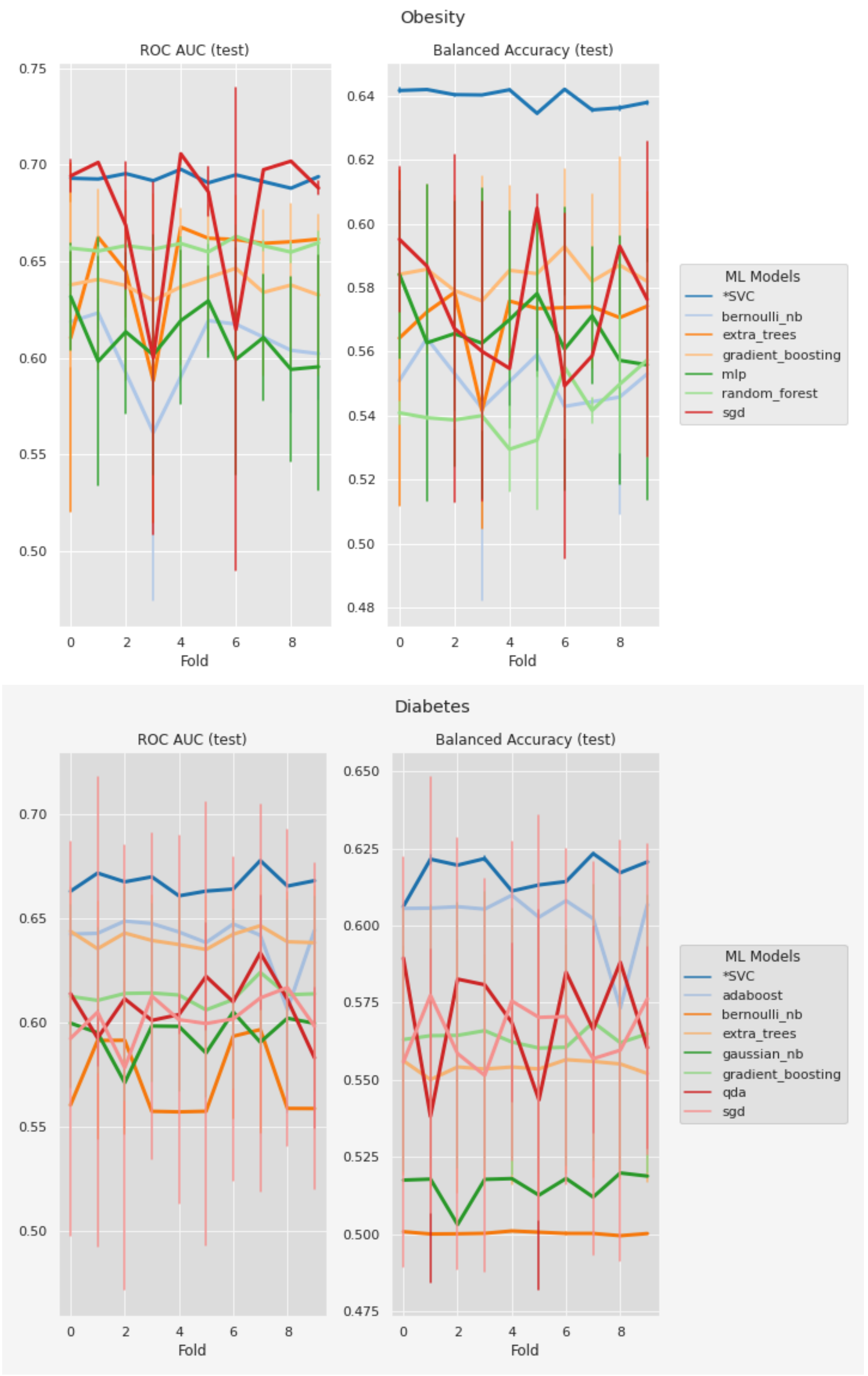
Obesity
ROC AUC (test)
Balanced Accuracy (test)
Fold
Fold
ML Models
*SVC
bernoulli_nb
extra_trees
gradient_boosting
mlp
random_forest
sgd
Diabetes
ROC AUC (test)
Balanced Accuracy (test)
Fold
Fold
ML Models
*SVC
adaboost
bernoulli_nb
extra_trees
gaussian_nb
gradient_boosting
qda
sgd

Supplementary table-4 Additional classification (CVM- and CVM+) performance metrics in the external validation dataset

A) Across the entire dataset, without excluding co-occurring CVMs					
**SPARE-models**	**ROC-AUC**	**Balanced Accuracy**	**Accuracy**	**Sensitivity**	**Specificity**
**Hypertension**	0.709	0.652	0.651	0.644	0.661
**Hyperlipidemia**	0.709	0.650	0.634	0.707	0.593
**Smoking**	0.633	0.595	0.597	0.591	0.599
**Obesity**	0.716	0.655	0.693	0.560	0.749
**Diabetes**	0.706	0.657	0.674	0.638	0.676
**B) In a subset of participants with no comorbidities (i.e., excluding participants with CVM+ in more than one CVM)**					
**SPARE-models**	**ROC-AUC**	**Balanced Accuracy**	**Accuracy**	**Sensitivity**	**Specificity**
**Hypertension**	0.698	0.637	0.643	0.547	0.728
**Hyperlipidemia**	0.708	0.639	0.665	0.592	0.686
**Smoking**	0.613	0.580	0.650	0.463	0.697
**Obesity**	0.698	0.625	0.724	0.478	0.772
**Diabetes**	0.692	0.673	0.759	0.585	0.761

4.2 Age-restricted modeling – Sensitivity analysis

The primary dataset and models for this study included all CU participants who were within 45-85 years of age. In addition to the primary model, additional models were tested for sensitivity analyses with age-restricted datasets. For each CVM, four separate models were trained and tested for the following age windows: 45-55 years, 55-65 years, 65-75 years, and 75-85 years of age. Effect sizes for SPARE-CVMs generated from age-specific models were tested and the results are shown in **Supplementary figure-9B**.

S5 – Supplementary Results

Supplementary figure-4: Individualized SPARE-CVMs

SPARE-CVM panels detect subtle, spatially distributed sMRI patterns that are not easily discernible through visual inspection. Illustration of the heterogeneous clinical profiles, neuroimaging presentations, and individualized SPARE- scores from samples across 3 age ranges (top: 45-55, middle: 55-65, and bottom row: 65-75 years).

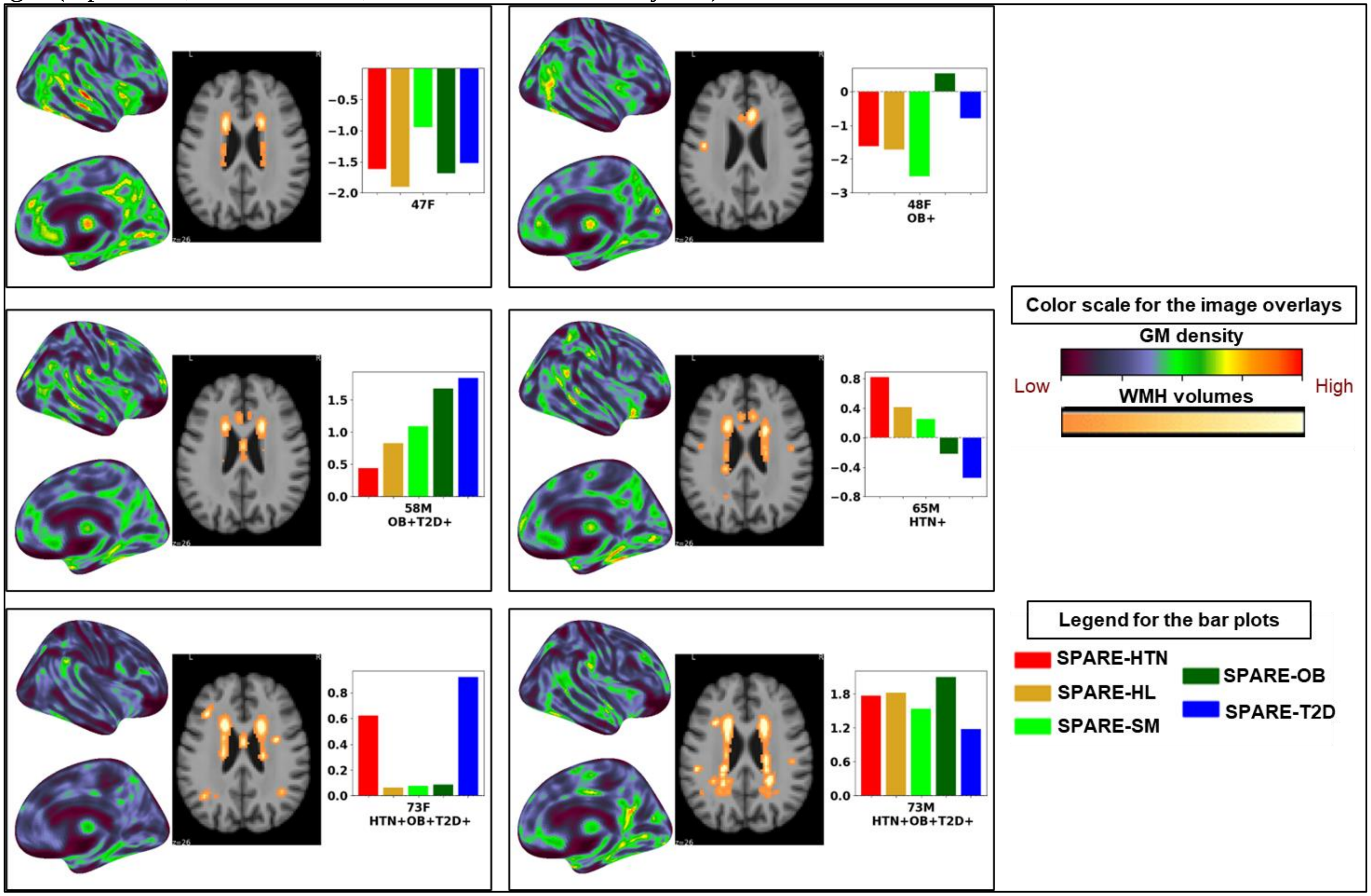

5.1 Spatial patterns of structural changes

Cortical GM patterns

Higher SPAREs for all CVMs were associated with a pattern of cortical atrophy in frontal GM regions including the anterior and posterior insula, the frontal and central opercular regions, and parts of the inferior frontal gyri, in parietal regions including the postcentral and supramarginal gyri, and temporal GM regions including the planum polare and planum temporale. In the frontal lobe, lower volumes in the middle frontal gyri, and the orbital gyri were associated with higher SPARE-HTN, SPARE-HL and SPARE-SM, the subcallosal area with higher SPARE-HTN, SPARE-HL and SPARE-OB, the posterior orbital gyri with higher SPARE-HTN and SPARE-T2D, and the supplementary motor cortex and the medial parts of the precentral and superior frontal gyri with SPARE-HL and SPARE-SM. In the parietal lobe, lower volumes in the angular gyrus were associated with higher SPARE-SM and SPARE-T2D. In the temporal lobe, lower volumes in the entorhinal area were associated with SPARE-HTN, SPARE-SM, SPARE-OB, and SPARE-T2D, and the superior temporal gyri with SPARE-SM and SPARE-T2D. In the occipital lobe, lower volumes in the lingual gyri were associated with higher SPARE-HTN, SPARE-SM and SPARE-T2D, and lower volumes in the cuneus and calcarine cortices were associated with higher SPARE-T2D. Relatively higher volumes in the middle occipital gyri, and the cingulate gyri were associated with higher SPARE-HL and SPARE-OB, the gyrus rectus with SPARE-HL, the supplementary motor cortex, the precuneus and the occipital gyri with higher SPARE-OB. Age was treated as a confounding variable for sMRI measures and SPARE-CVMs in multiple regression analyses. Hence the positive association noted here may be interpreted not as increasing volume, but rather as relatively preserved at older ages, suggesting potential resilience to atrophy in CVM+.

Deep GM patterns

Among deep GM structures, lower volumes in the accumbens area were associated with higher SPARE scores for all CVMs except SPARE-HL, the thalamus with higher SPARE-HL, SPARE-SM and SPARE-T2D, and the pallidum associated with SPARE-SM, SPARE-OB and SPARE-T2D. Relatively higher volumes in the hippocampus were associated with higher SPARE-HL and SPARE-OB, the putamen with SPARE-HTN and SPARE-HL, and the caudate nuclei with SPARE-HTN.

WM patterns

Lower volumes of WM regions were associated with SPARE-HL, SPARE-SM, SPARE-OB and SPARE-T2D. The strongest associations were observed between volumes of the anterior internal capsule and cerebellar WM and SPARE-HL, SPARE-SM and SPARE-T2D. In contrast, whereas higher WM volumes were associated with higher SPARE-HTN. We speculate that this positive association is driven by the presence of WMH which would contribute to the regional summary measures of WM extracted from T1-weighted sMRI.

WMH patterns

Higher SPARE-HTN was associated with larger WMH volumes across most of the sub-cortical and deep WM partitions, but not with WMH in the occipital lobe. Higher WMH volumes in deep WM structures were associated with higher SPARE-HL, SPARE-SM and SPARE-T2D.

Supplementary figure-5: Association between SPARE-CVM scores and MRI features

Regional associations between GM volumes and SPARE-CVMs from Main Figure 3 are visualized as heatmaps, displaying regression coefficients derived from two-sided multiple linear regression analyses (p < 0.001, Bonferroni corrected) after adjusting for age, sex, and ICV.

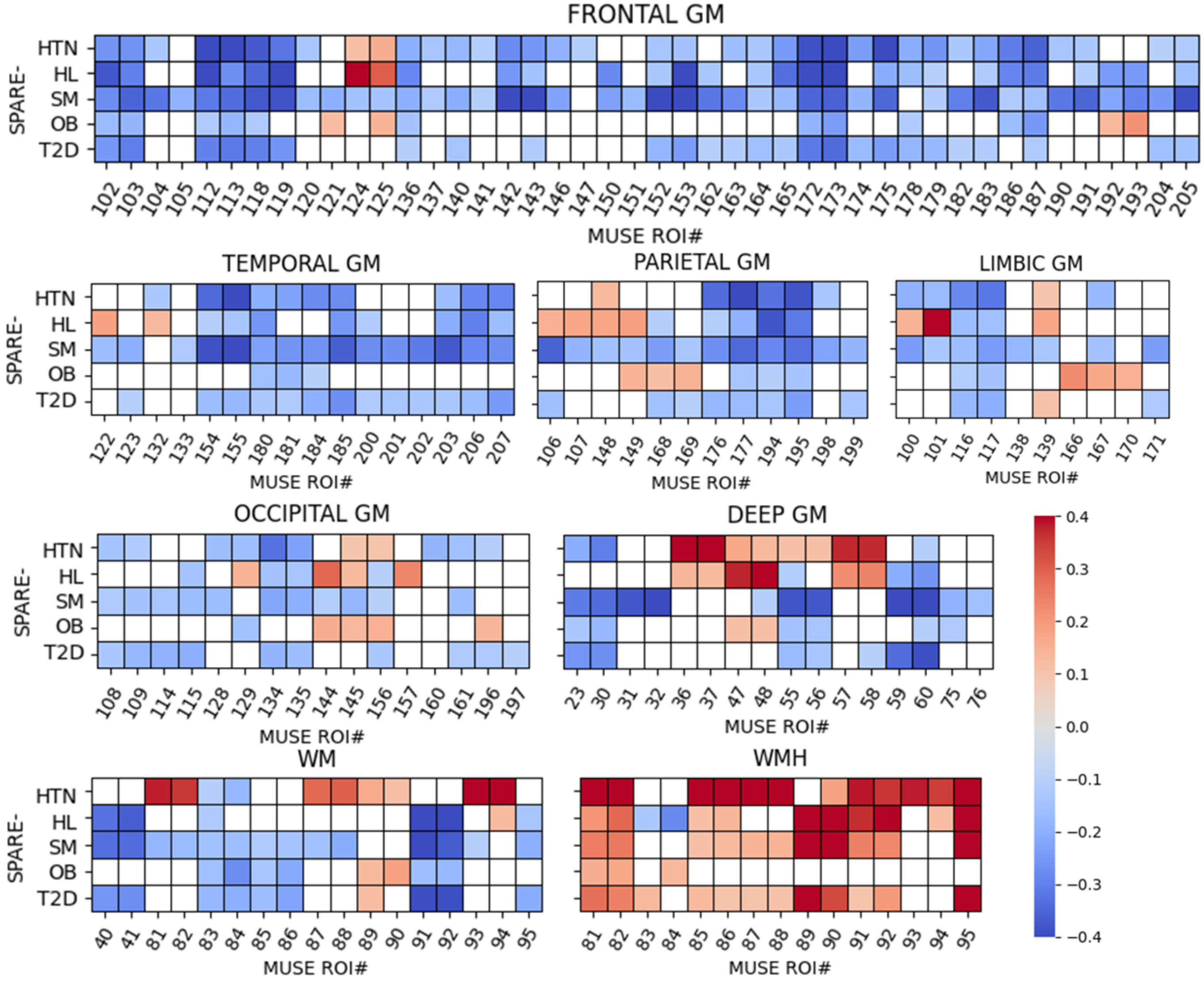

Supplementary figure-6: SPARE-CVM in the external validation cohort

(A) Heat-map of Cohen's d effect sizes of SPAREs (columns) for differentiating CVM+ from CVM- participants for each CVM (rows) in the validation cohort (UK Biobank v1.7, n =17, 096). Highlighted in blue are the effect sizes for the one-to-one correspondence between SPARE model and target CVM. (B) Results of logistic regression analyses where CVM status (+/-) was the outcome variable and the 5 SPARE-CVMs were the predictors, presented as odd-ratio (circle) and 95% confidence intervals (error bars). A unit increase in SPARE-CVM was associated with higher odds ratio of having a positive status in the target CVM. All associations were statistically significant with -log(p)>200. Abbreviations: HTN: Hypertension, HL: Hyperlipidemia, SM: Smoking, OB: Obesity, T2D: Type-2-Diabetes.

A)

B)

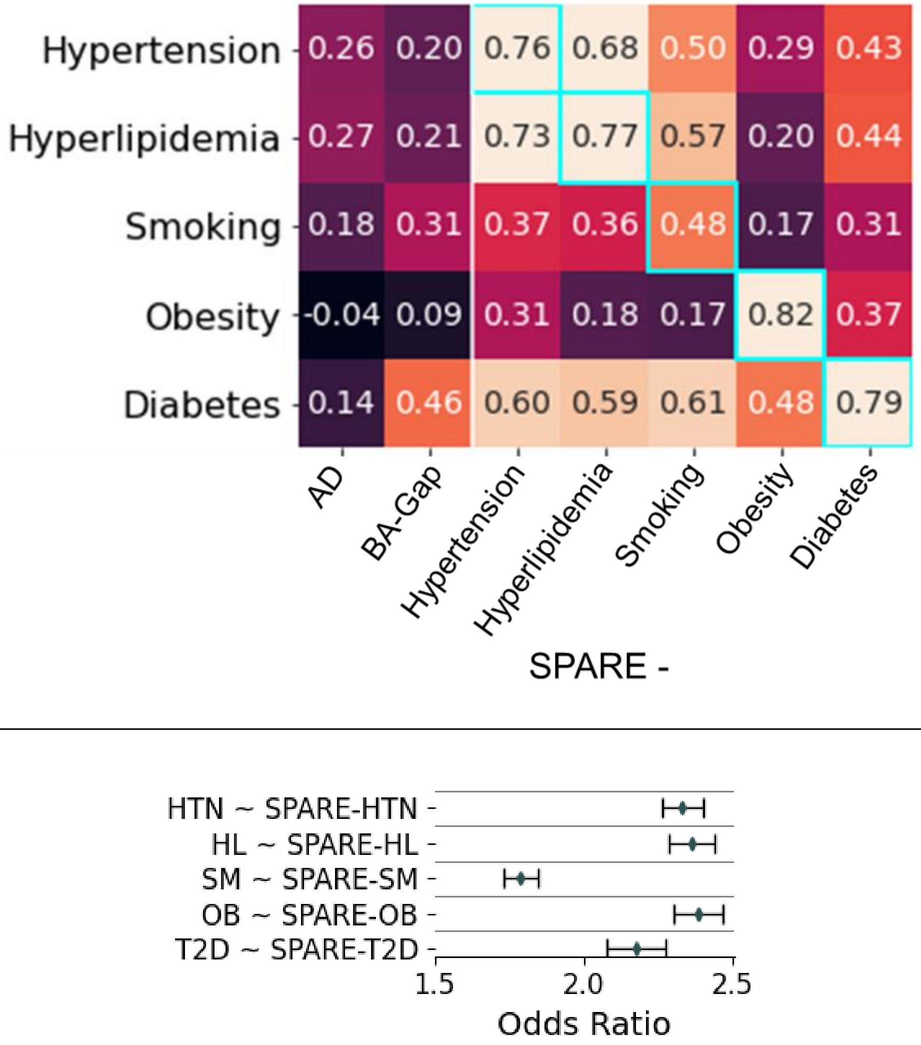

Supplementary figure-7: SPARE-CVMs are robust across demographic characteristics

A) Prevalence of CVM, and B) Distribution of SPAREs between CVM- and CVM+ participants across demographic and educational subgroups. Education levels were determined using years or categories of education across the studies. Level 2: <11 years, Level 3: 11-14 years, and Level 4: >14 years of education.

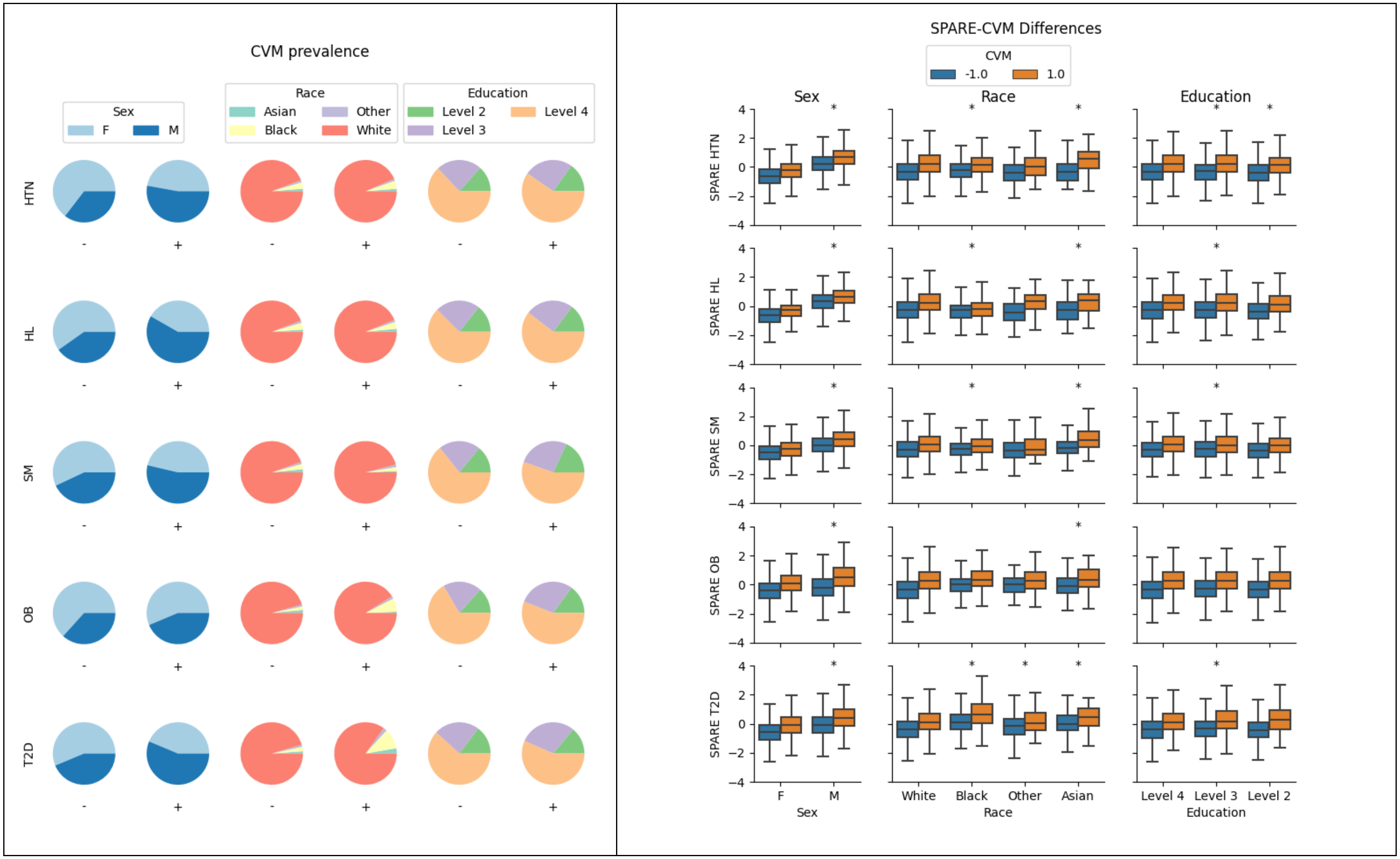

Supplementary figure-8: SPARE-CVMs complement established ML-based markers

Regression plots showing significant differences between CVM- and CVM+ observed in SPARE-AD and SPARE-BA-Gap in the ground truth dataset

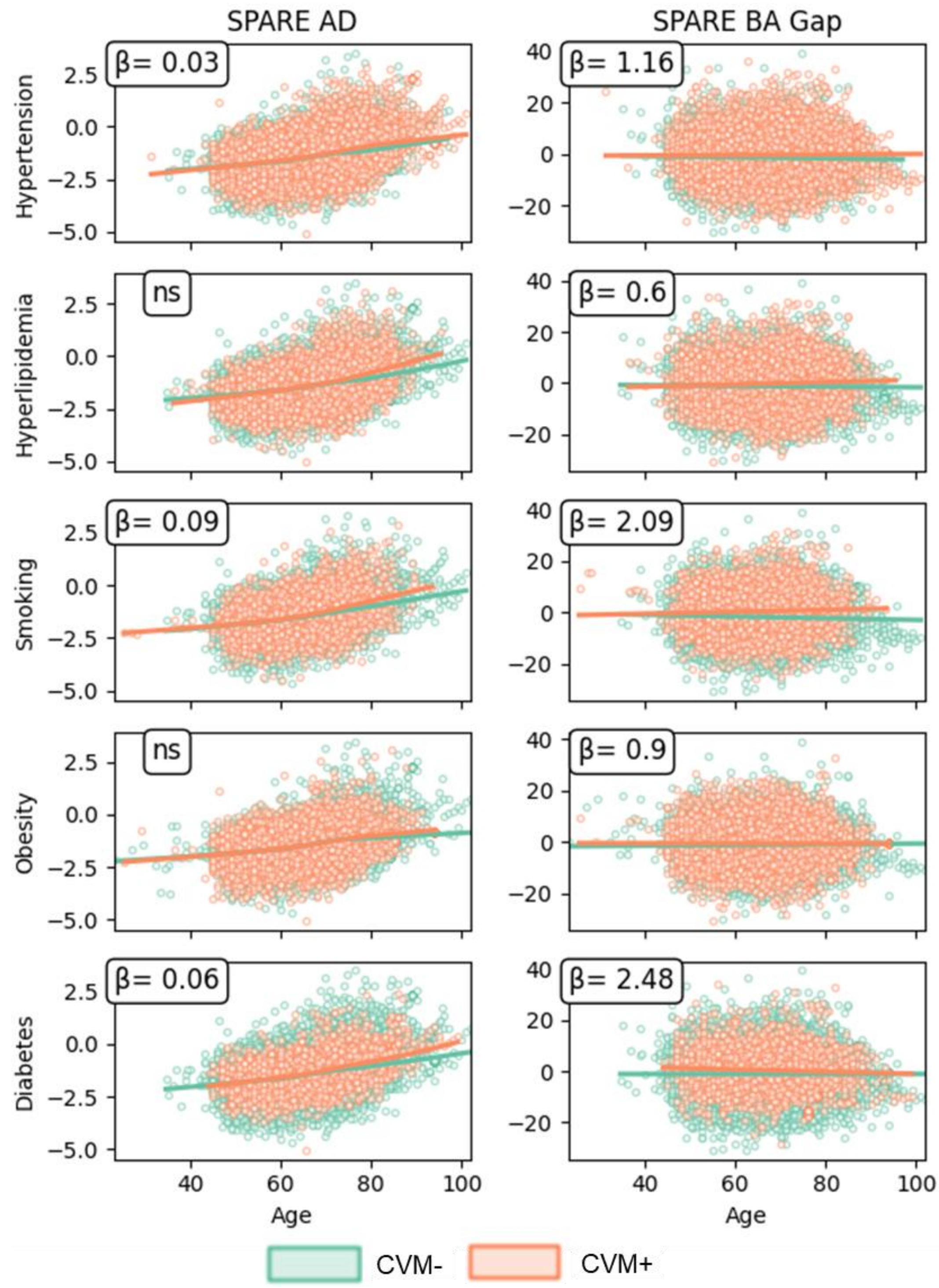

Supplementary figure-9: SPARE-CVMs across the age range

A) Distribution of SPAREs from the main study across the age windows. B) Effect sizes on new models trained on specific age windows (sensitivity analysis, Supplementary S 4)

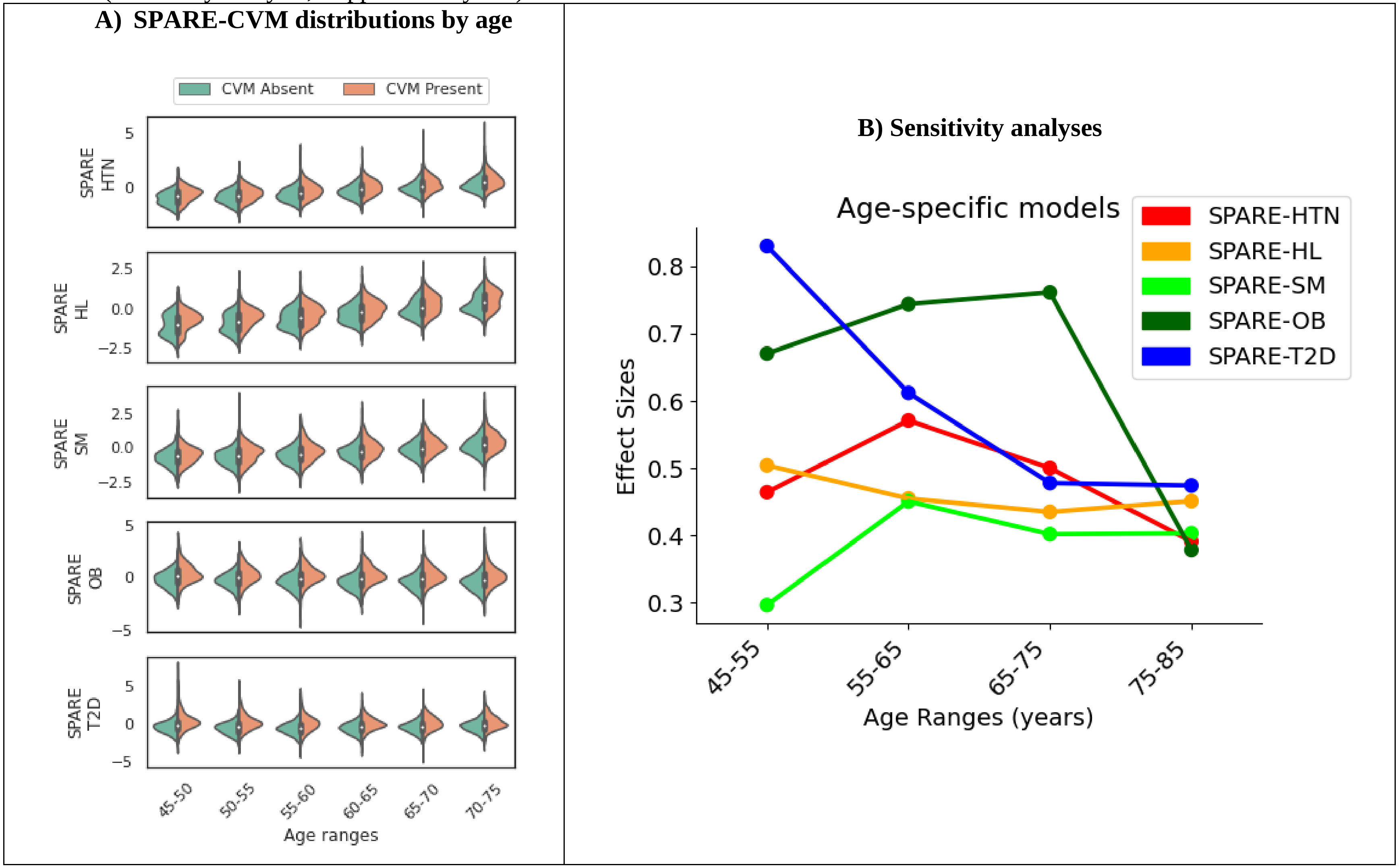

Supplementary figure-10: Influence of multimorbidity on SPARE-CVMs

A) 3 dimensional projections of the SPARE-CVMs to illustrate the influence of single- vs multi-morbidity in the corresponding SPARE scores. Each ellipsoid represents the SPARE-CVM scores closest to the mean for the corresponding combination of CVM statuses. Abbreviations: HTN – Hypertension; OB – Obesity; T2D – Type 2 Diabetes

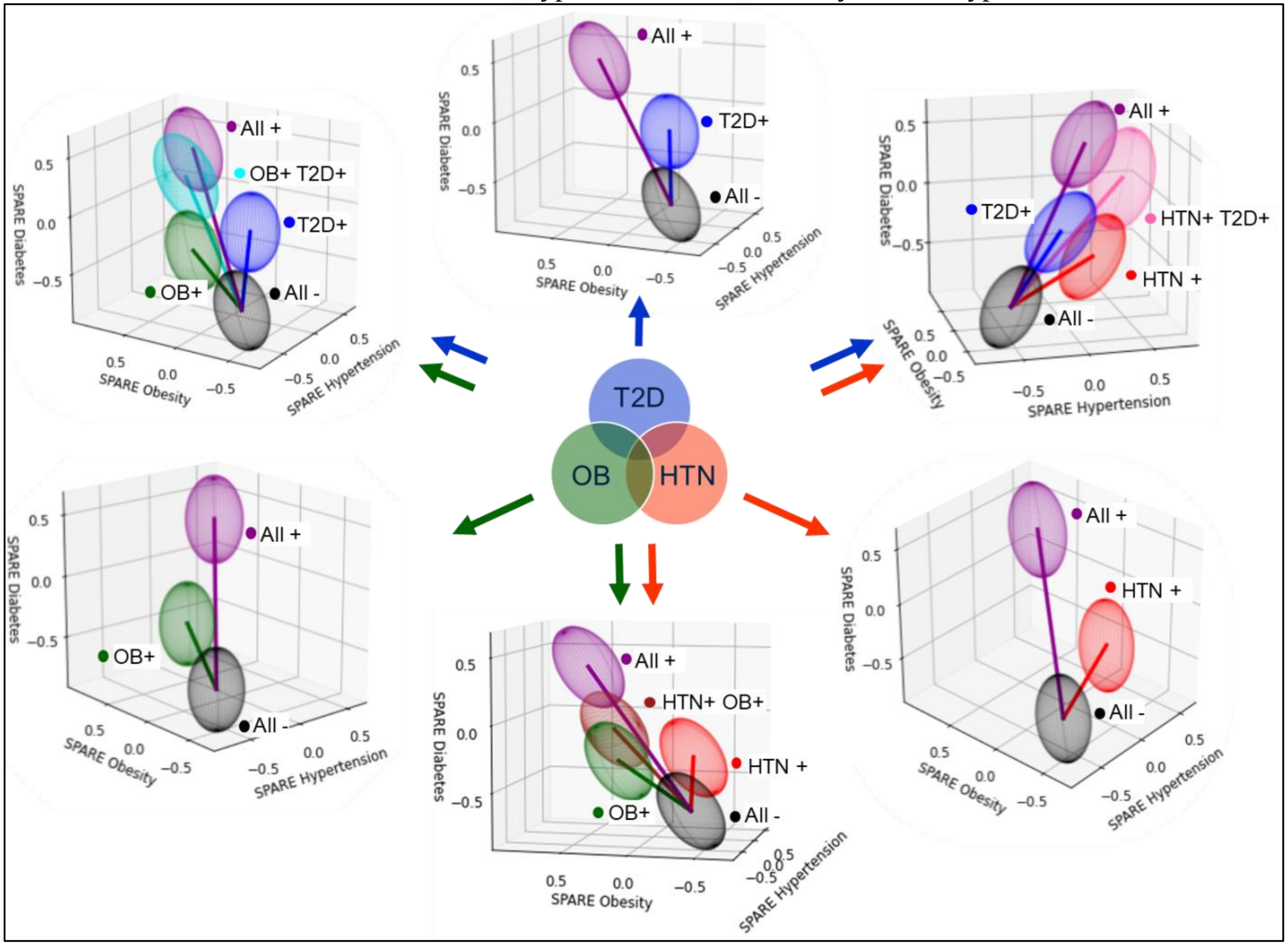

B) Cohen's d effect sizes of SPAREs (columns) for differentiating CVM+ from CVM- participants for each CVM (rows), recalculated from **Main Figure 4A** after excluding participants who had any co-occurring risk factors. Highlighted in blue are the effect sizes for the one-to-one correspondence between SPARE model and target CVM.

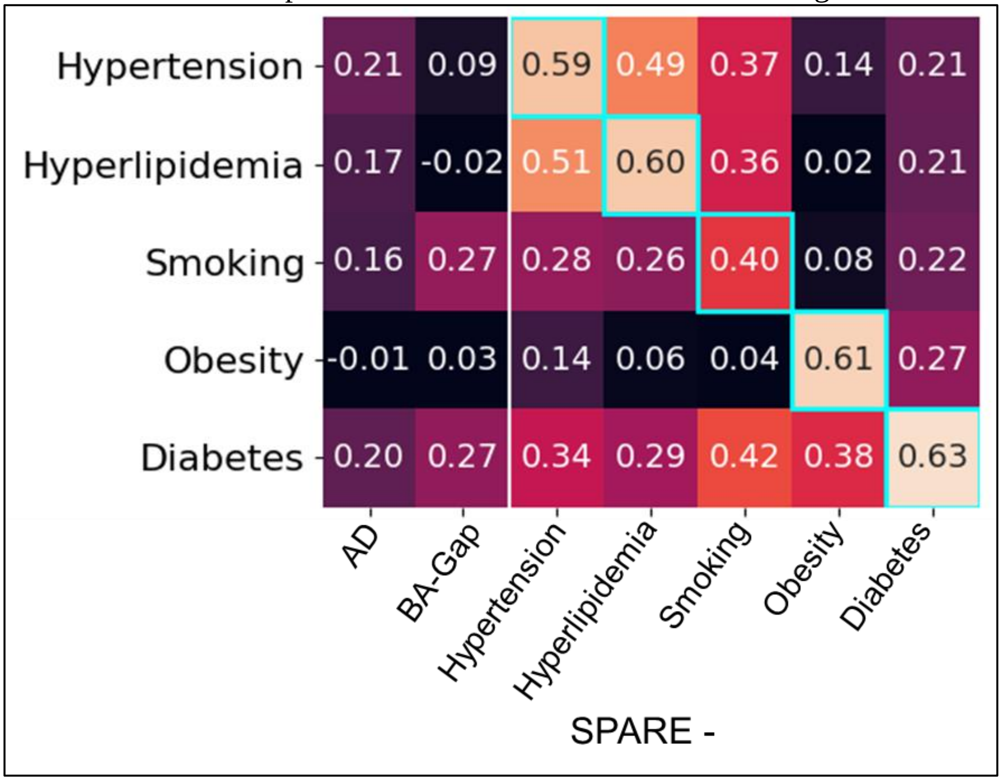

C) Results of logistic regression analyses demonstrating the specificity of SPARE- models to target CVM. Here, CVM status (+/-) was the outcome variable and the 5 SPARE-CVMs were the predictors. A unit increase in SPARE-CVM (rows) was associated with higher odds ratio of having a positive status in the target CVM (column).

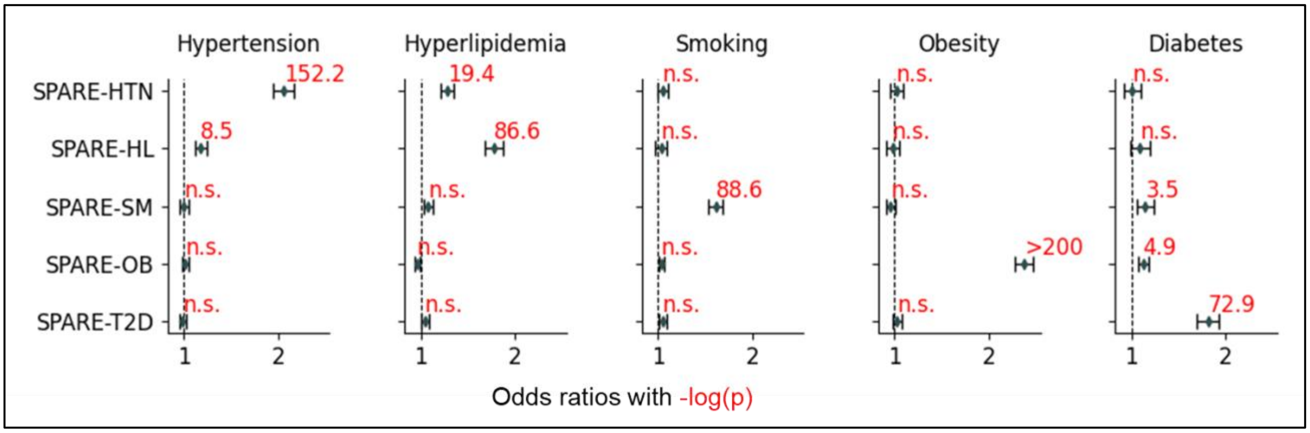

5.2 Associations with AD pathology

Sensitivity analyses for associations between SPARE-CVMs and AD pathology determined by amyloid deposition were carried out using statsmodels package in python. The effect of interactions between age, CVM status (+/-), and Aβ status (+/-) on SPARE-CVMs were evaluated using multiple regression models for each CVM.

Supplementary table-5: Distribution of age, sex, and CVMs in participants with amyloid Aβ data. Group differences between **Aβ- and Aβ+** were evaluated using two-sample t-tests for Age, and Chi-squared tests for categorical variables. Rows highlighted in bold indicate statistically significant group differences.

	Aβ **-**	Aβ +	**p-value**
N	292	115	
Age (SD)	**69.3 (8.0)**	**73.3 (7.0)**	**$p=2.8x10^{-6}$**
Sex – Female N (%)	157 (53.8%)	63 (54.8%)	p=0.94
**CVM prevalence**			
Hypertension N+/N-	118/115 (50.6 % HTN+)	38/48 (44.2% HTN+)	p=0.37
Hyperlipidemia N+/N-	107/112 (48.9 % HL+)	40/45 (47.1 % HL+)	p=0.88
Smoking N+/N-	55/107 (34.0 % SM+)	21/39 (35.0 % SM+)	p=1.0
Obesity N+/N-	**90/63 (58.8 % OB+)**	**15/46 (24.6 % OB+)**	**$p=1.24x10^{-6}$**
Diabetes N+/N-	26/204 (11.3 % T2D+)	6/83 (6.7% T2D+)	p=0.31

Supplementary figure-11. Influence of Aβ status, CVM status, and Age on SPARE-CVM scores
The results of the multivariate regression models testing the interaction between age, CVM status and Aβ status in predicting SPARE-CVMs.

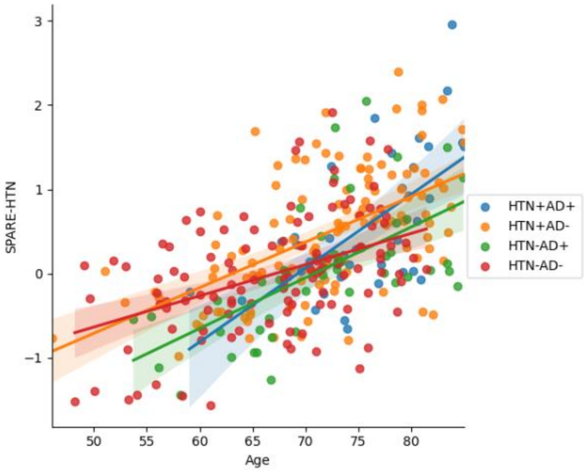

SPARE-HTN in Aβ+ individuals was lower in the younger ages of this sample (<70 years, p<0.01) and increased with advancing age (p<0.05), but did not reveal a significant interaction between HTN+ and Aβ+ status.

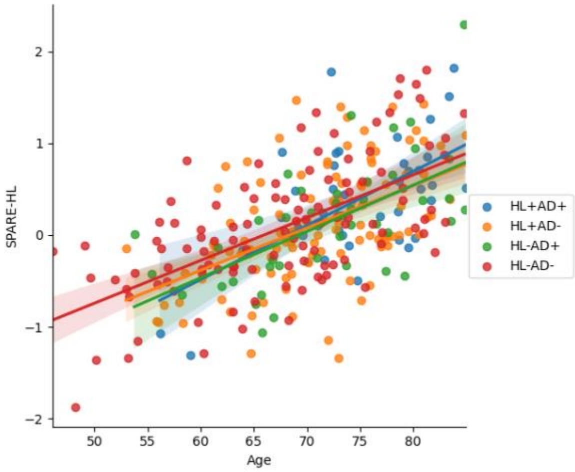

No significant associations between AβxHL status and SPARE-HL

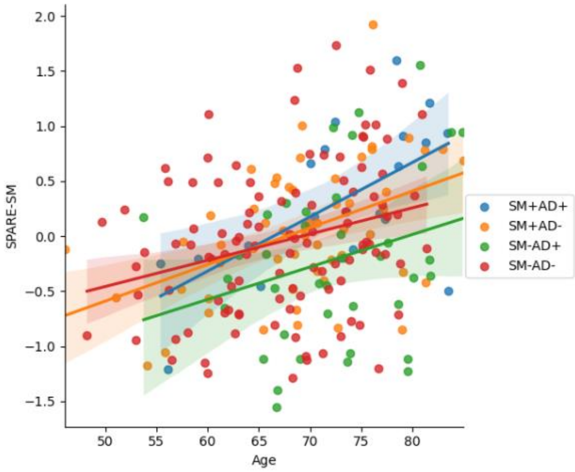

SPARE-SM was lower in SM- Aβ+ individuals (p<0.05) and higher in SM+ Aβ+ individuals (p<0.05), but did not show significant interactions between Aβ+ and age.

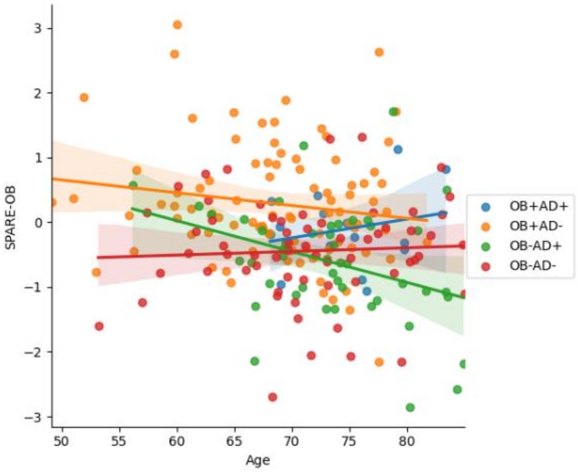	Significant age interactions were observed between OB status and Aβ+, where SPARE-OB decreased with age in OB- Aβ+ individuals (p<0.05) and trended towards an increase with age in OB+ Aβ+ individuals (p=0.06).
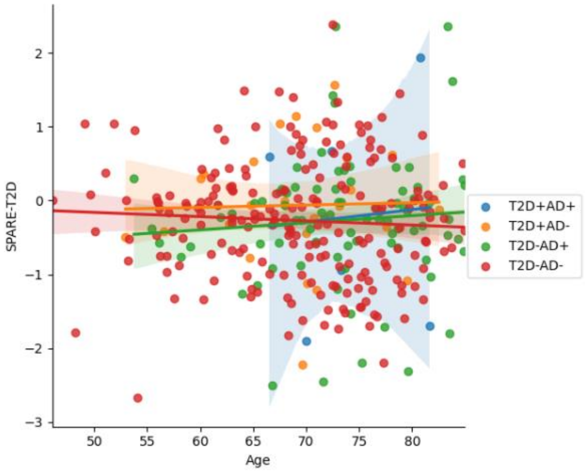	No significant associations between AβxT2D status and SPARE-T2D

5.3 Associations between cognitive performance and SPARE-CVM

To have a meaningful investigation of cognition across the dataset, we chose only the cognitive tests that were implemented in multiple studies using the standardized protocols. We separated the cognitive investigations in UK Biobank from the rest of the dataset to minimize biased results due to the study's large sample size and additionally, because procedures in UK Biobank differed from the standardized cognitive protocols. For example, the digit symbol substitution test (DSST) was conducted over half the duration (60 seconds) in UKBioBank as opposed to the standard 120 seconds [31]. All statistical modeling for associations between cognition and phenotypes were performed using statsmodels package in python. Multivariate linear regressions models predicting cognitive performance using SPARE-CVMs, while adjusting for the confounding covariates of study, age, sex, and number of years of education, were implemented on the rest of the dataset excluding UKBioBank cohort.

Supplementary table-6: Availability of cognitive test scores

Only participants whose education was encoded in number of years were included in this investigation. Abbreviations- DSST: Digit symbol substitution test; TMT-A/B: Trail making test A/B; MMSE: Mini-mental state examination; MOCA: Montreal cognitive assessment, DSST: Digit symbol substitution test.

	**Number of participants**				
**Study**	DSST	TMT_A	TMT_B	MOCA	MMSE
**ADNI**	-	321	318	291	297
**AIBL**	133	-	-	-	135
**BIOCARD**	22	-	-	-	21
**BLSA**	39	915	912	-	803
**CARDIA**	812	-	-	169	-
**OASIS**	36	36	36	-	41
**PENN**	19	183	183	124	187
**WRAP**	39	39	38	-	39
**Total**	1100	1494	1487	584	1523

Supplementary figure-12: Association between cognitive performance and SPARE-CVMs or CVM status.

The results of the multivariate regression models predicting cognitive performance using SPARE-CVMs or CVM status, while adjusting for the confounding covariates of study, age, sex, and number of years of education, are shown below. Regression coefficients are represented by circles, with error bars indicating 95% confidence intervals. SPARE-CVM scores were adjusted for potential confounders, namely age, sex, and DLICV, prior to the regression analysis. Participants without CVM (CVM-) were the reference group in the regression models for diagnostic status. Abbreviations- DSST: Digit symbol substitution test; TMT-A/B: Trail making test A/B; MMSE: Mini-mental state examination; MOCA: Montreal cognitive assessment. False discovery rate corrected p-values are indicated by: ^ $p = 0.05$, * $p< 0.05$, ** $p < 0.01$, † $p< 0.001$, and †† $p<<0.001$. Associations with p-values >0.05 are not marked.

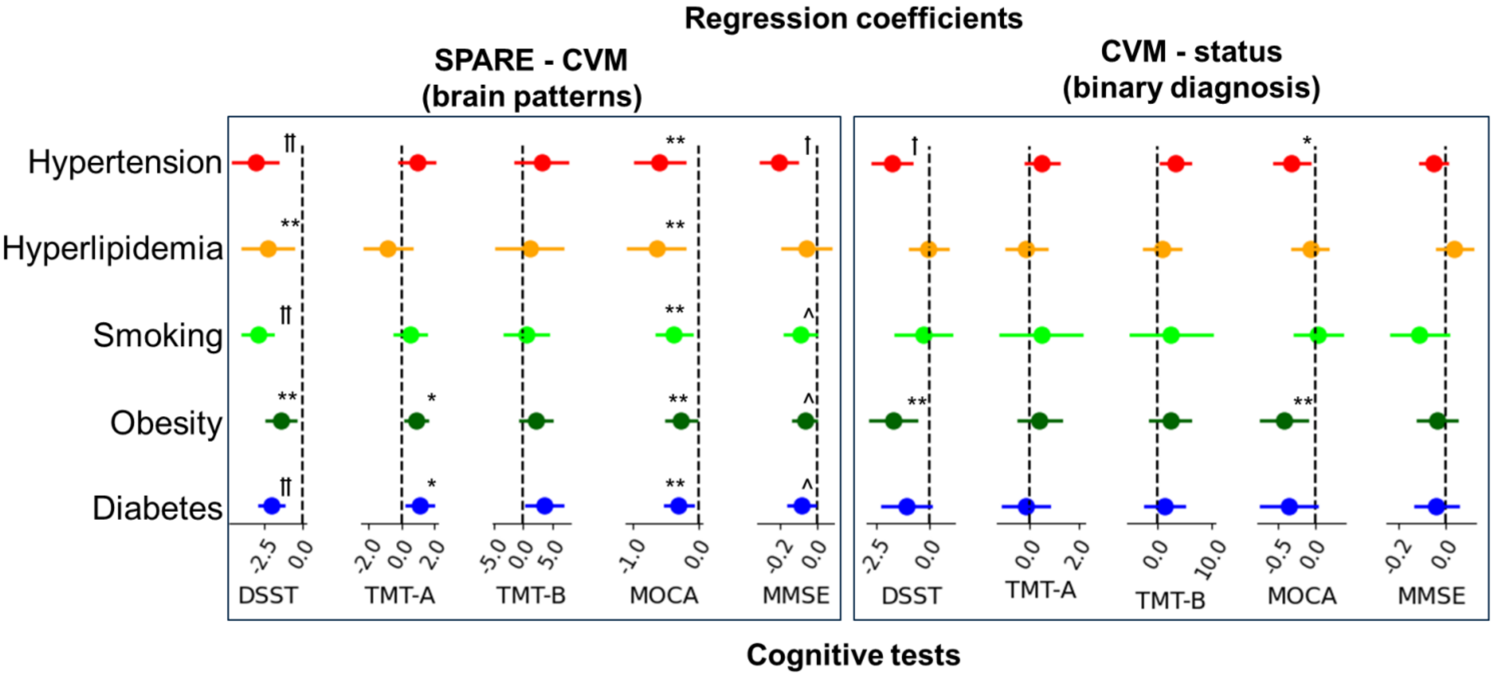

5.4 Effect of ROI harmonization on SPARE-CVMs

We performed Analysis of Variance (ANOVA) to evaluate the impact of site differences on SPARE-CVMs trained using Harmonized and Unharmonized MUSE ROI volumes. By comparing linear models with and without 'site' as a covariate, we found that harmonization drastically reduced site-related variability in SPARE-CVMs, as evidenced by lower F-values (**Supplementary figure-13**). In contrast, Unharmonized SPARE-CVMs exhibited substantial site effects, limiting their generalizability beyond the original study sites despite effective CVM classification within those sites.

Supplementary figure-13: Analysis of Variance (ANOVA) evaluation of site effects
F-values from ANOVA comparing the effect of 'site' in SPARE-CVMs trained using harmonized and unharmonized MUSE ROI volumes are shown below. Site effects have a substantial influence on SPARE-CVMs derived using unharmonized MUSE ROI volumes. This comparison included models built on all training participants.

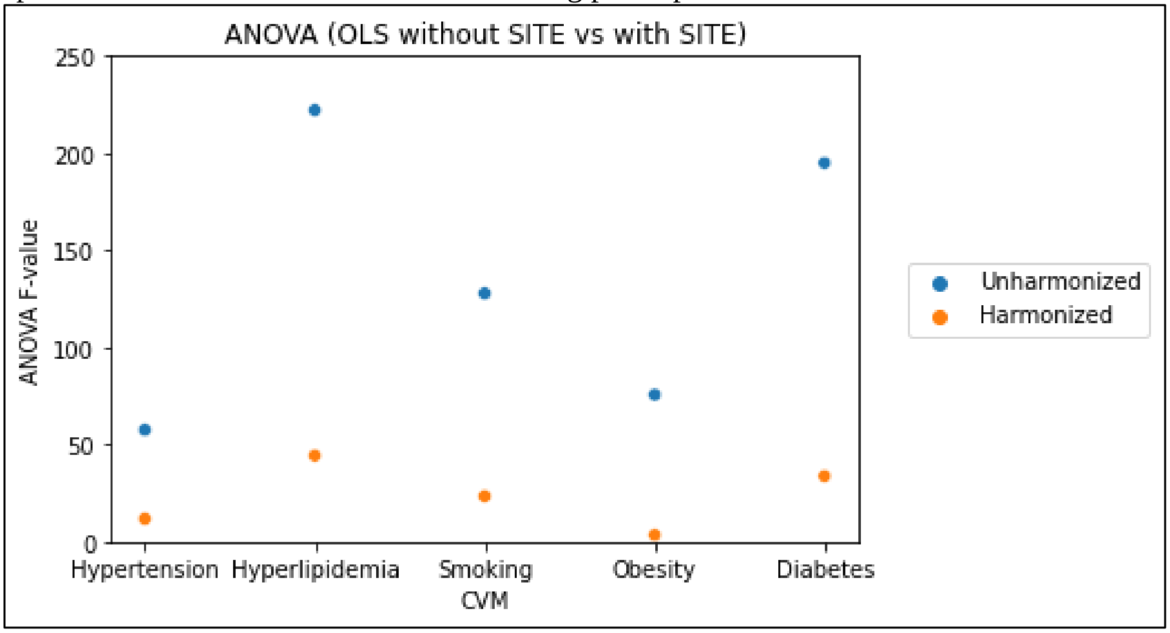

Study references

1. Habes, M., et al., *The Brain Chart of Aging: Machine-learning analytics reveals links between brain aging, white matter disease, amyloid burden, and cognition in the iSTAGING consortium of 10,216 harmonized MR scans.* Alzheimers Dement, 2021. **17**(1): p. 89-102.
2. Weiner, M.W., et al., *The Alzheimer's disease neuroimaging initiative: progress report and future plans.* Alzheimer's & Dementia, 2010. **6**(3): p. 202-211. e7.
3. Jack Jr, C.R., et al., *The Alzheimer's disease neuroimaging initiative (ADNI): MRI methods.* Journal of Magnetic Resonance Imaging: An Official Journal of the International Society for Magnetic Resonance in Medicine, 2008. **27**(4): p. 685-691.
4. Pietrzak, R.H., et al., *Trajectories of memory decline in preclinical Alzheimer's disease: results from the Australian Imaging, Biomarkers and Lifestyle Flagship Study of Ageing.* Neurobiology of aging, 2015. **36**(3): p. 1231-1238.
5. Albert, M., et al., *Cognitive changes preceding clinical symptom onset of mild cognitive impairment and relationship to ApoE genotype.* Current Alzheimer Research, 2014. **11**(8): p. 773-784.
6. Ferrucci, L., *The Baltimore Longitudinal Study of Aging (BLSA): a 50-year-long journey and plans for the future*. 2008, Oxford University Press. p. 1416-1419.
7. Cermakova, P., et al., *Subclinical cardiac dysfunction and brain health in midlife: CARDIA (Coronary Artery Risk Development in Young Adults) brain magnetic resonance imaging substudy.* Journal of the American Heart Association, 2017. **6**(12): p. e006750.
8. Marcus, D.S., et al., *Open Access Series of Imaging Studies (OASIS): cross-sectional MRI data in young, middle aged, nondemented, and demented older adults.* Journal of cognitive neuroscience, 2007. **19**(9): p. 1498-1507.
9. Wolk, D.A., et al., *Amyloid imaging in Alzheimer's disease: comparison of florbetapir and Pittsburgh compound-B positron emission tomography.* Journal of Neurology, Neurosurgery & Psychiatry, 2012. **83**(9): p. 923-926.
10. Alfaro-Almagro, F., et al., *Image processing and Quality Control for the first 10,000 brain imaging datasets from UK Biobank.* Neuroimage, 2018. **166**: p. 400-424.
11. Resnick, S.M., et al., *Postmenopausal hormone therapy and regional brain volumes: the WHIMS-MRI Study.* Neurology, 2009. **72**(2): p. 135-142.
12. Johnson, S.C., et al., *The Wisconsin Registry for Alzheimer's Prevention: a review of findings and current directions.* Alzheimer's & Dementia: Diagnosis, Assessment & Disease Monitoring, 2018. **10**: p. 130-142.
13. *ADNI MRI Protocols*. [cited 2024 October]; Available from: https://adni.loni.usc.edu/methods/documents/mri-protocols/.
14. Yates, P.A., et al., *Incidence of cerebral microbleeds in preclinical Alzheimer disease.* Neurology, 2014. **82**(14): p. 1266-73.
15. Newton, P., et al., *Regional White Matter Hyperintensities and Alzheimer's Disease Biomarkers Among Older Adults with Normal Cognition and Mild Cognitive Impairment.* J Alzheimers Dis, 2023. **92**(1): p. 323-339.
16. *CARDIA BRAIN MRI SUBSTUDY*. [cited 2024 October]; Available from: https://www.cardia.dopm.uab.edu/images/more/pdf/mooy25/chapter12.pdf.
17. *UK Biobank Brain Imaging - Acquisition Protocol*. [cited 2024 October 18]; Available from: https://www.fmrib.ox.ac.uk/ukbiobank/protocol/V4_23092014.pdf.

18. Pomponio, R., et al., *Harmonization of large MRI datasets for the analysis of brain imaging patterns throughout the lifespan.* Neuroimage, 2020. **208**: p. 116450.
19. *CARDIA BRAIN MRI SUBSTUDY*. Available from: https://www.cardia.dopm.uab.edu/images/more/pdf/mooy25/chapter12.pdf.
20. Kraut, M.A., et al., *The impact of magnetic resonance imaging-detected white matter hyperintensities on longitudinal changes in regional cerebral blood flow.* J Cereb Blood Flow Metab, 2008. **28**(1): p. 190-7.
21. Resnick, S.M., et al., *One-year age changes in MRI brain volumes in older adults.* Cereb Cortex, 2000. **10**(5): p. 464-72.
22. Moghekar, A., et al., *Cerebral white matter disease is associated with Alzheimer pathology in a prospective cohort.* Alzheimers Dement, 2012. **8**(5 Suppl): p. S71-7.
23. Davatzikos, C., et al., *Longitudinal progression of Alzheimer's-like patterns of atrophy in normal older adults: the SPARE-AD index.* Brain, 2009. **132**(Pt 8): p. 2026-35.
24. Hwang, G., et al., *Disentangling Alzheimer's disease neurodegeneration from typical brain ageing using machine learning.* Brain Communications, 2023. **4**(3).
25. Chen, Y., et al., *Detecting brain structural changes as biomarker from magnetic resonance images using a local feature based SVM approach.* J Neurosci Methods, 2014. **221**: p. 22-31.
26. Steardo, L., Jr., et al., *Application of Support Vector Machine on fMRI Data as Biomarkers in Schizophrenia Diagnosis: A Systematic Review.* Front Psychiatry, 2020. **11**: p. 588.
27. Noteboom, S., et al., *Evaluation of machine learning-based classification of clinical impairment and prediction of clinical worsening in multiple sclerosis.* J Neurol, 2024.
28. Pedregosa, F., et al., *Scikit-learn: Machine Learning in Python.* Journal of Machine Learning Research, 2023. **12**(85): p. 2825-2830.
29. Cawley, G.C. and N.L.C. Talbot, *On Over-fitting in Model Selection and Subsequent Selection Bias in Performance Evaluation.* J. Mach. Learn. Res., 2010. **11**: p. 2079–2107.
30. Feurer, M., et al., *Efficient and robust automated machine learning.* Advances in neural information processing systems, 2015. **28**.
31. Cockcroft, K., et al., *A cross-cultural comparison between South African and British students on the Wechsler Adult Intelligence Scales Third Edition (WAIS-III).* Front Psychol, 2015. **6**: p. 297.
