# Supplementary material for: Machine learning reveals distinct neuroanatomical signatures of cardiovascular and metabolic diseases in cognitively unimpaired individuals
Source: Nat Commun. 2025 Mar 19;16:2724. doi: 10.1038/s41467-025-57867-7 (PMC11923046; doi:10.1038/s41467-025-57867-7)
Supplement: Supplementary file 2 — Reporting Summary [file 41467_2025_57867_MOESM2_ESM.pdf]

nature portfolio

Corresponding author(s): Sindhuja Tirumalai Govindarajan, Christos Davatzikos

Last updated by author(s): 12/20/2024

Reporting Summary

Nature Portfolio wishes to improve the reproducibility of the work that we publish. This form provides structure for consistency and transparency in reporting. For further information on Nature Portfolio policies, see our Editorial Policies and the Editorial Policy Checklist.

Statistics

For all statistical analyses, confirm that the following items are present in the figure legend, table legend, main text, or Methods section.

n/a	Confirmed	
☐	☒	The exact sample size (n) for each experimental group/condition, given as a discrete number and unit of measurement
☐	☒	A statement on whether measurements were taken from distinct samples or whether the same sample was measured repeatedly
☐	☒	The statistical test(s) used AND whether they are one- or two-sided<br>*Only common tests should be described solely by name; describe more complex techniques in the Methods section.*
☐	☒	A description of all covariates tested
☐	☒	A description of any assumptions or corrections, such as tests of normality and adjustment for multiple comparisons
☐	☒	A full description of the statistical parameters including central tendency (e.g. means) or other basic estimates (e.g. regression coefficient) AND variation (e.g. standard deviation) or associated estimates of uncertainty (e.g. confidence intervals)
☐	☒	For null hypothesis testing, the test statistic (e.g. F, t, r) with confidence intervals, effect sizes, degrees of freedom and P value noted<br>*Give P values as exact values whenever suitable.*
☒	☐	For Bayesian analysis, information on the choice of priors and Markov chain Monte Carlo settings
☒	☐	For hierarchical and complex designs, identification of the appropriate level for tests and full reporting of outcomes
☐	☒	Estimates of effect sizes (e.g. Cohen's d, Pearson's r), indicating how they were calculated

Our web collection on statistics for biologists contains articles on many of the points above.

Software and code

Policy information about availability of computer code

Data collection	No new data was collected for this study. Software packages used for processing the data from existing cohorts are listed below:<br>MUSE: https://github.com/CBICA/MUSE<br>DeepMRSeg: https://github.com/CBICA/DeepMRSeg<br>MRISnapshot: https://github.com/CBICA/MRISnapshot<br>Combat-GAM: https://github.com/CBICA/NiChart_Harmonize<br>The individual modules have been packaged in a comprehensive NiChart:Neuro Imaging Chart of AI-based Imaging Biomarkers platform (https://neuroimagingchart.com/)
Data analysis	Modeling and analyses utilized Python (version 3.8.1) and the models were developed using scikit-learn (version 1.3.2). We used several other Python and R libraries to support data analysis and visualization, including pandas (version 1.5.3), statsmodels (version 0.13.2), numpy (version 1.22.4), matplotlib (version 3.5.13), seaborn (version 0.12.2), scipy(1.7.3), ggplot2 (version 3.4.4), and venn (version 1.11). Python scripts for data processing and the machine learning model are available on GitHub: https://github.com/CBICA/NiChart_Project and https://github.com/stgovindarajan/spare_cvm_score

For manuscripts utilizing custom algorithms or software that are central to the research but not yet described in published literature, software must be made available to editors and reviewers. We strongly encourage code deposition in a community repository (e.g. GitHub). See the Nature Portfolio guidelines for submitting code & software for further information.

Data

Policy information about availability of data

All manuscripts must include a data availability statement. This statement should provide the following information, where applicable:

- Accession codes, unique identifiers, or web links for publicly available datasets
- A description of any restrictions on data availability
- For clinical datasets or third party data, please ensure that the statement adheres to our policy

SPARE-CVM indices derived in this study have been uploaded as Supplementary Data in the Source Data file. Original imaging and clinical data used in this study were obtained through data-sharing agreements from the following ten individual studies: Alzheimer's Disease Neuroimaging Initiative (ADNI, Australian Imaging, Biomarker and Lifestyle Flagship Study of Ageing (AIBL), Biomarkers of Cognitive Decline Among Normal Individuals (BIOCARD), Baltimore Longitudinal Study of Aging (BLSA), Coronary Artery Risk Development in Young Adults (CARDIA), Open Access Series of Imaging Studies (OASIS), Penn Memory Center (PENN), UK Biobank (UKBB), Women's Health Initiative Memory Study (WHIMS), and Wisconsin Registry for Alzheimer's Prevention (WRAP). The data-sharing agreements do not include permission for us to share the data further. Investigators must apply to the source data providers to access additional data and match their subject IDs to those used in this study under the current protocol (primarily for UKBB). Data from ADNI and AIBL are available from the Imaging and Data Archive database (https://ida.loni.usc.ed) upon registration and compliance with the data usage agreement. Data from the UKBB are available upon request from the UKBB website (https://www.ukbiobank.ac.uk/). Data from the BLSA study are available upon request at https://www. blsa.nih.gov/how-apply. Data from the OASIS study are available upon request at https://www.oasis-brains.org/. Data requests for BIOCARD, PENN, WRAP, CARDIA, and WHIMS datasets should be directed to M.S.A., D.A.W., S.C.J., L.J.L., and M.A.E., respectively. Upon obtaining access to the source data, investigators can match our derived SPARE-CVM indices to the rest of the data from these studies. Further assistance in matching the R-indices can be requested from the corresponding last author, C.D., at Christos.Davatzikos@pennmedicine.upenn.edu, with responses typically provided within 2 weeks. Moreover, we are actively following protocols to upload our derived measures to the UKBB and ADNI websites, making them directly accessible to investigators who obtain access to those studies.

Research involving human participants, their data, or biological material

Policy information about studies with human participants or human data. See also policy information about sex, gender (identity/presentation), and sexual orientation and race, ethnicity and racism.

Reporting on sex and gender	The data was obtained from existing cohorts. The sex distributions from the different studies are reported in Table 1. We also performed sex-specific sensitivity analysis to evaluate the robustness of our markers (Figure S-7).
Reporting on race, ethnicity, or other socially relevant groupings	The data was obtained from existing cohorts and study-reported race distributions are presented in Table 1. We reported race and ethnicity information on all the cohorts grouped as White, Black, Asian, or Other categories whenever available. We also report the distribution of participants with different levels of education attainment (number or years categorized as <11 years, 11-14 years, and >14 years). We also reported the distribution of clinical factors and performed a sensitivity analysis to evaluate the robustness of our markers within race and educational categories (Figure S-7). Educational attainment was used as a confounding variable when evaluating the association of cognitive performance with brain measures.
Population characteristics	Participants in the study were 37,098 individuals with a mean age of 65. They included 17,065 males and 20,033 females. All participants presented with no known cognitive impairment as defined by study-specific criteria. Based on reported data, the racial composition was 34,887 White, 941 Black, 504 Asian, and 461 with other multiracial or minority ethnicities. Reported educational attainment distributions were 21,802 with >14 years, 8555 with 11-14 years, and 4027 with <11 years of educational attainment. Composition of risk factor prevalence was 15137 with hypertension, 10589 with hyperlipidemia, 7447 smokers, 6993 with obesity, and 2403 with type-2 diabetes. More details on the population characterization can be found in the Methods section.
Recruitment	The data was obtained from existing cohorts. No additional participants were recruited for this study.
Ethics oversight	The University of Pennsylvania institutional review board approved this research study. Participants provided written informed consent to the corresponding source studies.

Note that full information on the approval of the study protocol must also be provided in the manuscript.

Field-specific reporting

Please select the one below that is the best fit for your research. If you are not sure, read the appropriate sections before making your selection.

☒ Life sciences ☐ Behavioural & social sciences ☐ Ecological, evolutionary & environmental sciences

For a reference copy of the document with all sections, see nature.com/documents/nr-reporting-summary-flat.pdf

Life sciences study design

All studies must disclose on these points even when the disclosure is negative.

Sample size	The data was obtained from ten existing cohorts. No sample size calculation was carried out. We considered all subjects satisfying the inclusion/exclusion and imaging quality control criteria described in the manuscript. We included 20,000 participants for training and 17,096 participants for validating the model. More details on the study population can be found in the manuscript.

Data exclusions	We excluded data from the cohorts based on the exclusion criteria described in the manuscript. Briefly, participants with cognitive impairment, dementia, or other neurological conditions were excluded. Participants with incomplete sMRI data or whose sMRI data failed QC were also excluded from the study.
Replication	Model outputs were validated in an unseen dataset in this study. Researchers interested in replicating study findings are able to do so by following the methods described in the manuscript or running the code in our GitHub repository. Several of the cohorts used in this study including ADNI, AIBL, and OASIS are open to the public. Other cohort data may be requested through institutional data-sharing agreements. Study models and normative distributions are also available in the NiChart:Neuro Imaging Chart of AI-based Imaging Biomarkers platform (https://neuroimagingchart.com/). NiChart allows researchers across the globe to upload their own study data, process structural MRI for deriving volumetric features, harmonize said features to iSTAGING dataset, and predict SPARE-CVMs.
Randomization	We utilized a consistent random seed to perform randomized stratified sampling, shuffling training and testing splits for repeated cross-validation experiments in order to train the machine learning models.
Blinding	No blinding was carried out in this study as it is a retrospective analysis of existing cohorts with no defined clinical outcome.

Reporting for specific materials, systems and methods

We require information from authors about some types of materials, experimental systems and methods used in many studies. Here, indicate whether each material, system or method listed is relevant to your study. If you are not sure if a list item applies to your research, read the appropriate section before selecting a response.

Materials & experimental systems

n/a	Involved in the study
☒	☐ Antibodies
☒	☐ Eukaryotic cell lines
☒	☐ Palaeontology and archaeology
☒	☐ Animals and other organisms
☒	☐ Clinical data
☒	☐ Dual use research of concern
☒	☐ Plants

Methods

n/a	Involved in the study
☒	☐ ChIP-seq
☒	☐ Flow cytometry
☐	☒ MRI-based neuroimaging

Plants

Seed stocks	Not applicable to the study
Novel plant genotypes	Not applicable to the study
Authentication	Not applicable to the study

Magnetic resonance imaging

Experimental design

Design type	Retrospective cohort study
Design specifications	This is a retrospective cohort study on data obtained from existing cohorts. All data were available at study onset.
Behavioral performance measures	Clinical and cognitive performance measures were obtained from the primary studies.

Acquisition

Imaging type(s)	Structural MRI (T1, T2, FLAIR)
Field strength	1.5 and 3 Tesla
Sequence & imaging parameters	All MRI were obtained from the primary cohort studies. Information on cohort-specific sequences and imaging parameters is provided in Table S1. No additional imaging was performed in this study.
Area of acquisition	Whole brain

Diffusion MRI ☐ Used ☒ Not used

Preprocessing

Preprocessing software	All image processing tools used in this study are publicly available on the NiChart platform and the other packages on our GitHub repository: https://github.com/CBICA/.<br>MUSE: https://github.com/CBICA/MUSE<br>DeepMRSeg: https://github.com/CBICA/DeepMRSeg<br>MRISnapshot: https://github.com/CBICA/MRISnapshot<br>Combat-GAM: https://github.com/CBICA/NiChart_Harmonize
Normalization	Study analyses used region of interest volume measures based on the multi-atlas MUSE segmentations. MUSE derives consensus segmentation labels from an ensemble of warping algorithms applied to multiple atlases. Derived MUSE volumes were standardized across study collection sites using Combat-GAM harmonization.
Normalization template	MNI152 template was used for visualizing the SPARE-CVM associations with ROI volumes.
Noise and artifact removal	Raw images and segmentation masks underwent a two-step semi-automated quality control procedure to exclude participants with severe artifacts. The multi-atlas MUSE method is a consensus labeling approach that uses deformable registration algorithms to reduce the impact of missing mutual information, and is hence robust to noise and imaging artifacts.<br>Doshi et al., MUSE: MUlti-atlas region Segmentation utilizing Ensembles of registration algorithms and parameters, and locally optimal atlas selection. Neuroimage, 2016.
Volume censoring	Not applicable to sMRI data used in this study.

Statistical modeling & inference

Model type and settings	To test associations between each SPARE-CVM and sMRI measures, we fitted separate multiple linear regression models for each ROI adjusting for age, sex, and intracranial volume.
Effect(s) tested	Associations between SPARE-CVMs and sMRI volumes.

Specify type of analysis: ☐ Whole brain ☒ ROI-based ☐ Both

Anatomical location(s)	Grey and white matter tissue volumes in ROIs segmented using MUSE and WMH volumes summarized within the lobar and deep WM ROIs. The list of all ROIs is provided in Table S2.
Statistic type for inference<br>(See Eklund et al. 2016)	ROI-level inference. Coefficient (β) for ROI associations with SPARE-CVMs from the multiple regression models adjusted for age, sex, and intracranial volume are reported (Figure 4 and S-5).
Correction	Multiple linear regression p-values were corrected for multiple comparisons using the Bonferroni method using an alpha of 0.001.

Models & analysis

n/a	Involved in the study
☒	☐ Functional and/or effective connectivity
☒	☐ Graph analysis
☐	☒ Multivariate modeling or predictive analysis

Multivariate modeling and predictive analysis	Supervised classifiers were trained independently for each of the five CVMs to separate input features of CVM+ and CVM- . Input features were the volumes mentioned above. Linear support vector classifiers were trained using a nested cross-validation approach with stratified sampling. Models were evaluated using the balanced accuracy metric. Additional validation of the model configuration included comparisons with other classifiers using balanced accuracy and area under the receiver operating curve, as detailed in Appendix S4.
